# Electrolyte Engineering toward Rational Electrode–Electrolyte Interfacial Designs for Metal Batteries

**DOI:** 10.1002/advs.202516870

**Published:** 2025-11-17

**Authors:** Yunlong Yang, Xuchao Yang, Xinle Liu, Lei Zhao, Dongxia Luo, Zhihua Hai, Yuantong Guo, Hongquan Niu, Fen Ran

**Affiliations:** ^1^ State Key Laboratory of Advanced Processing and Recycling of Non‐ferrous Metals School of Material Science and Engineering Lanzhou University of Technology Lanzhou 730050 China

**Keywords:** electrolyte additives, metal batteries, philic‐phobic interface, solid electrolyte interface, superwetting

## Abstract

Lithium, zinc, sodium, potassium, and magnesium metal batteries have emerged as the core direction of next‐generation energy storage technologies due to their ultrahigh theoretical capacities. However, the uncontrollable dendrite growth and unstable solid electrolyte interface of metal anodes during cycling lead to battery short‐circuiting, capacity fading, and safety hazards, severely hindering their commercialization process. Further design of excellent electrode‐electrolyte interfaces requires precisely identifying and addressing related scientific challenges from multiple dimensions. Specifically, this review focuses on the evolution of the solid electrolyte interphase with philic‐phobic properties on the surface of metal electrodes and discusses key influencing factors governing its evolution, including electrolyte additives and artificial solid electerolyte interphase. The research progress in solid electrolyte interface with philic‐phobic properties regulation using electrolyte additives and other methods in recent years is systematically summarized. Additionally, the influence mechanisms of electrode–electrolyte interface design on battery performance under extreme environments for lithium metal batteries and novel metal batteries are emphatically discussed. Future research needs to deeply explore the micro‐mechanisms and develop more efficient materials and technologies to further improve battery performance and meet the growing demands of the energy storage field.

## Graphical abstract

This contribution systematically discusses the philic‐phobic interface design, working principles, and regulatory strategies for interfacial issues in metal batteries. It also elaborates on the underlying mechanisms, implementation strategies, and their impacts on battery performance, while emphatically exploring the influence mechanisms of electrode–electrolyte interface design on metal battery performance under extreme environments.

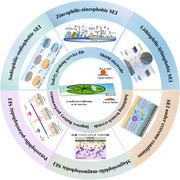

## 1 Introduction

As technology continues to advance and applications grow increasingly specialized, there is a rapidly rising demand for batteries with novel functions and properties tailored to meet diverse specific requirements. As an indispensable component of modern society, batteries find extensive use across a wide spectrum of fields—from powering portable electronic devices and electric vehicles to supporting large‐scale renewable energy storage systems. The performance of batteries directly influences the development and application of related technologies; consequently, enhancing battery performance has become a focal point for researchers worldwide.^[^ ^1^, ^2^, ^3^, ^4^ ^]^ Within battery systems, the interfacial characteristics between electrodes and electrolytes play a pivotal role in determining overall performance.^[^ ^2^, ^5^ ^]^

Metal batteries (such as lithium, sodium, potassium, etc.) are regarded as ideal candidates for future energy storage devices due to their ultrahigh theoretical capacities.^[^ ^6^ ^]^ However, uncontrollable dendrite growth and unstable solid electrolyte interface (SEI) on metal anodes during cycling lead to battery short‐circuiting, capacity fading, and safety hazards, posing significant threats to battery safety.^[^ ^7^, ^8^, ^9^, ^10^ ^]^ For instance, lithium dendrites formed in lithium metal batteries cause the loss of active lithium, drastically reducing the battery's Coulombic efficiency (CE) and shortening its cycle life.^[^ ^8^, ^11^ ^]^ These issues become more pronounced under fast charging conditions, severely hindering the practical application and commercialization process of metal batteries. As a nanoscale film formed by the reaction between the electrolyte and metal anode, the SEI's chemical composition, mechanical properties, and ion transport characteristics directly determine the cycle life and rate performance of batteries. Strategies to enhance battery cycle stability and safety include adding functional electrolyte additives, regulating electrolyte composition, and constructing artificial interfacial layers.^[^ ^8^, ^12^, ^13^ ^]^

As we all know, within the realm of metal‐based batteries, the affinity and repellency characteristics between electrolytes and metals—acting as core parameters governing interfacial wettability, charge transfer kinetics, and reaction selectivity—have gradually emerged as a focal point of research in the field.^[^ ^14^, ^15^ ^]^ On the one hand, the metal‐affine property facilitates the uniform deposition of metal ions and suppresses the nucleation and growth of dendrites, primarily by lowering the metal nucleation energy barrier at the electrode surface. On the other hand, the metal‐repellent property can establish a physical barrier to minimize direct contact between the metal and electrolyte; concurrently, it guides the ordered vertical growth of dendrites, thereby preventing lateral dendrite propagation and penetration of the separator.^[^ ^16^, ^17^ ^]^

Philic‐phobic interface design enables the development of high‐rate and high‐safety batteries by optimizing ion transport kinetics, regulating metal ion deposition behavior, and enhancing interfacial stability.^[^ ^24^, ^25^, ^26^, ^27^, ^28^ ^]^ Such philic‐phobic interface design can be achieved via strategies including electrolyte additives and the construction of artificial SEI, among others. In recent years, the design and optimization of electrolyte additives have been proven effective strategies for regulating the structure of the SEI.^[^ ^29^ ^]^ By incorporating functional additives (e.g., fluorinated carbonates, ionic liquids, and nanoparticles), the film‐forming kinetics, chemical composition, and physical properties of the SEI can be precisely modulated. However, current research still confronts multiple challenges: inadequate compatibility between additives and high‐voltage cathodes, ambiguous failure mechanisms of the SEI under a wide temperature range, and high costs hindering the large‐scale application of additives.^[^ ^30^ ^]^ Furthermore, achieving more accurate and efficient interfacial regulation to meet the performance requirements of diverse battery systems under complex operating conditions remains a critical challenge in current studies. Moreover, certain artificial SEIs with philic‐phobic interface can also be designed and constructed, such as those based on polyethyleneimine, metal‐organic frameworks, and polyethylene glycol chains, and others.^[^ ^31^, ^32^, ^33^, ^34^ ^]^

**Figure 1:**
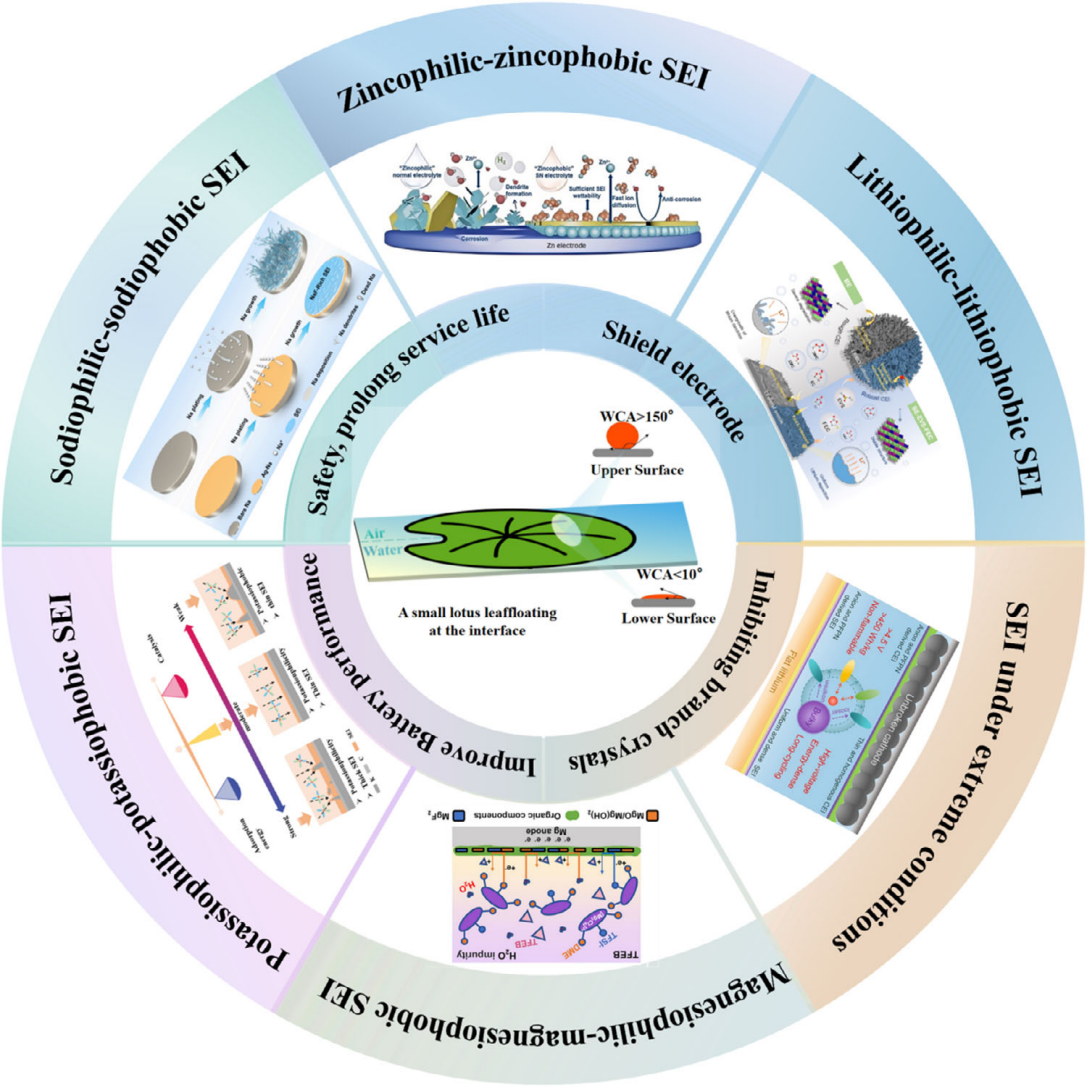
Overview of the topics covered in this review.^[^ ^18^, ^19^, ^20^, ^21^, ^22^, ^23^ ^]^

This review systematically summarizes the formation mechanism of the philic‐phobic interface design in metal batteries, the working principles and regulatory strategies, and discusses the opportunities and challenges associated with interfacial issues in metal batteries. It also elaborates in detail on the underlying mechanisms, implementation strategies, and their impacts on battery performance (**Figure** **1**). Furthermore, focusing on the characteristics of different metal systems (e.g., Li/Zn/Na/K/Mg), the review analyzes the commonalities and differences in how additives regulate the SEI. By systematically summarizing and deeply analyzing recent research advances, this work aims to provide a solid theoretical basis and innovative technical insights for the development of high‐performance batteries.

## 2 Mechanism of SEI Formation

### 2.1 The Formation Process of SEI

**Figure 2:**
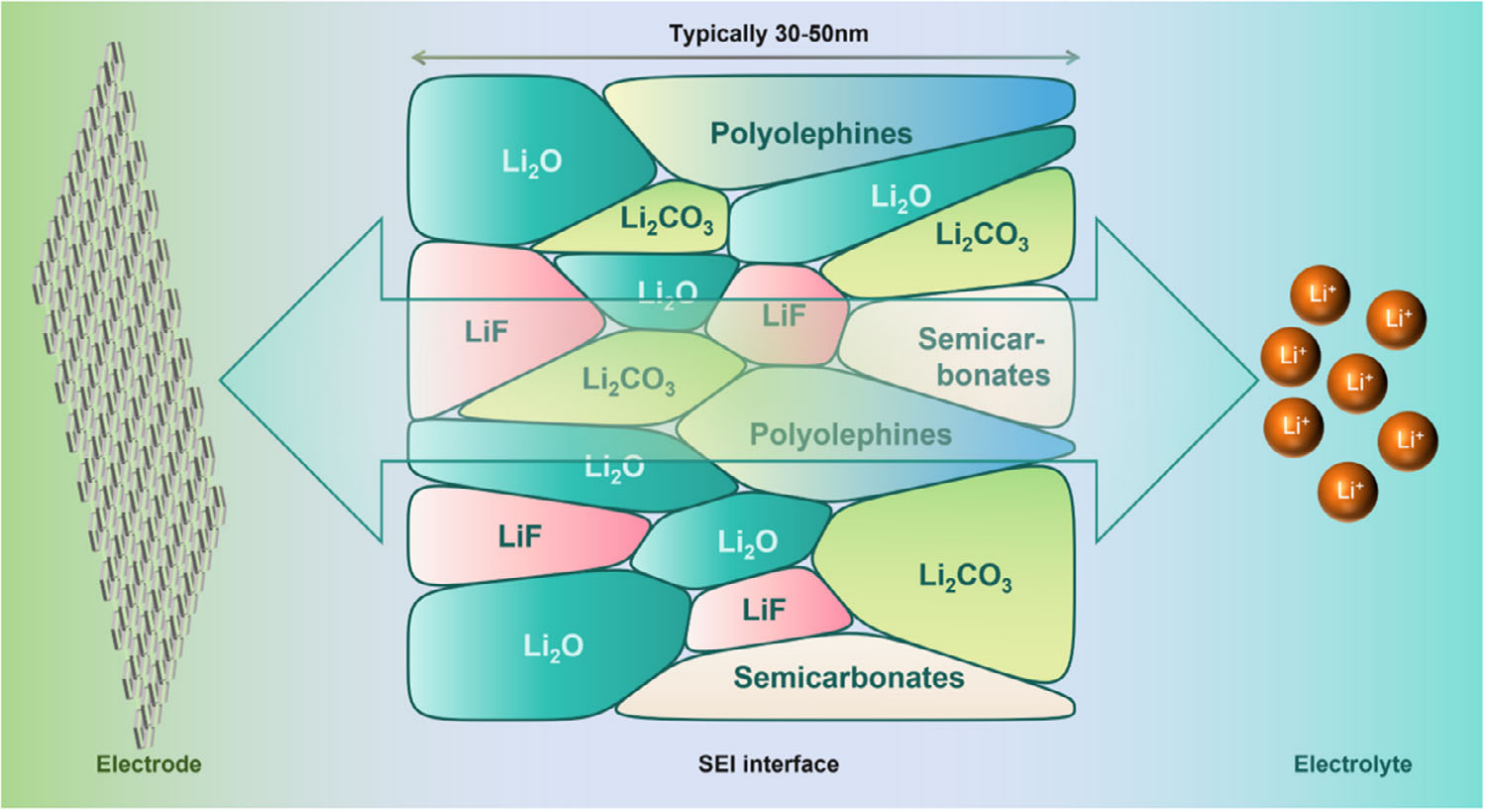
Schematic diagram of SEI formation in lithium batteries.

When the battery is first charged, the electrode material begins a complex series of chemical reactions with the electrolyte,^[^ ^30^ ^]^ resulting from the extremely high chemical activity of lithium metal anodes. Solvent molecules, such as carbonate compounds, will undergo reduction reactions on the surface of lithium metal to form some initial reduction products. In this process, the lithium salts in the electrolyte, and especially the anionic part, may react with the lithium metal to form the corresponding compound. These initial reaction products begin to deposit on the electrode surface, forming the prototype SEI.^[^ ^35^, ^36^ ^]^ As the charging process continues, more electrolyte components are consumed, and the resulting reaction products continue to accumulate on the electrode surface. These products interact and aggregate to gradually form a continuous film, named the SEI layer. During this process, the thickness of the SEI layer increases, and structure gradually becomes complex. In **Figure** **2**, a schematic diagram of SEI formation is shown using lithium batteries as an example. Some of the reaction products of small molecules may further polymerize or react with other substances to form larger molecular structures or more stable compounds. At the same time, the components of the SEI layer also diffuse and rearrange, enabling the structure of the SEI layer denser and more stable. At this stage, the reaction rate step by step slows down until the SEI layer reaches a certain thickness and stability, at which time the growth of the SEI layer tends to be balanced. In the subsequent charging and discharging cycles of the battery, the SEI layer is not static.^[^ ^37^ ^]^ Although SEI overall structure remains relatively stable, at the microscopic level, the SEI layer constantly repairs and adjusts by itself. When the battery is progressively discharging, the Li^+^ removing from the anode, and the SEI layer may be damaged or changed to a certain extent. During the charging process, Li^+^ is deposited on the anode, and certain components in electrolyte may react with the anode surface again, supplementing and repairing the SEI layer to maintain its integrity and functionality. This dynamic balancing process ensures that the SEI layer can continue to play a role during the battery cycles, protecting the anode and maintaining the stable performance of the battery.^[^ ^38^, ^39^ ^]^

Beyond lithium metal batteries, the formation process of the SEI layer in other metal‐based batteries—including those based on zinc, sodium, potassium, and magnesium—also conforms to the general pattern of “first‐charge‐triggered reaction, product deposition, and dynamic repair”.^[^ ^40^, ^41^ ^]^However, due to variations in the properties of metal ions (e.g., ionic radius, charge) and electrolyte systems (organic vs aqueous), their SEI layers differ significantly in terms of composition, formation pathways, and protective mechanisms.^[^ ^6^, ^42^ ^]^

In zinc metal batteries, aqueous systems are the most prevalent. The reduction potential of H_2_O in aqueous electrolytes is lower than that of most organic solvents. During the initial charging process, H_2_O undergoes primary reactions on the zinc electrode surface, accompanied by the hydrolysis of Zn^2^⁺; these processes collectively drive the formation of the SEI layer.^[^ ^43^, ^44^, ^45^ ^]^ Hydroxide ions (OH^−^) generated by the reduction of H_2_O combine with Zn^2^⁺ to form zinc hydroxide (Zn(OH)_2_). Dissolved carbon dioxide (CO_2_) in the electrolyte reacts with Zn^2^⁺ to produce zinc carbonate (ZnCO_3_). When the electrolyte contains sulfate anions (e.g., from ZnSO_4_), a basic zinc sulfate (ZnSO_4_·3Zn(OH)_2_·H_2_O, abbreviated as ZHS) is further formed. Since most SEI products in aqueous environments are highly soluble, their formation process depends on the “in situ deposition‐dynamic equilibrium” mechanism. ZHS exhibits low solubility and can tightly adhere to the zinc electrode surface, forming a continuous SEI layer. Meanwhile, Zn(OH)_2_ and ZnCO_3_ function as auxiliary components to fill the pores.^[^ ^46^, ^47^ ^]^ During cycling, zinc electrodes are prone to dendrite formation due to the growth of Zn^2^⁺ along grain boundaries. The ZHS within the SEI can guide Zn^2^⁺ flux through uniformly distributed nanochannels, thereby inhibiting dendrite formation. Additionally, the SEI can physically isolate H_2_O from metallic zinc, mitigating hydrogen evolution side reactions and electrode corrosion, which in turn ensures the cycling stability of zinc batteries.

Sodium metal has a slightly lower electronegativity (0.93) than lithium (0.98), resulting in marginally weaker chemical activity.^[^ ^48^ ^]^ However, the ionic radius of Na⁺ (1.02 Å) is larger than that of Li⁺ (0.76 Å), which endows the formation of its SEI with unique characteristics.^[^ ^49^ ^]^ During the initial charging process, ether‐based (e.g., 1,2‐dimethoxyethane, DME) or carbonate‐based (e.g., ethylene carbonate, EC) solvents undergo reduction reactions on the sodium electrode surface, producing primary inorganic products such as Na_2_CO_3_ and Na_2_O. Concurrently, anions from sodium salts (e.g., NaPF_6_, NaFSI) in the electrolyte react with metallic sodium, generating corrosion‐resistant inorganic components including NaF and Na_2_SO_3_.^[^ ^50^, ^51^, ^52^, ^53^, ^54^ ^]^ These products are deposited on the electrode surface, thereby forming a SEI layer.

Potassium metal exhibits the lowest electronegativity (0.82) and the highest chemical reactivity. During the initial charging process, the electrolyte undergoes more vigorous decomposition reactions, which serve as the core driving force for SEI formation.^[^ ^6^, ^55^ ^]^ Carbonate‐based solvents (e.g., diethyl carbonate) undergo rapid reduction to form inorganic products such as K_2_CO_3_ and K_2_O. Fluoride‐containing potassium salts (e.g., KPF_6_) preferentially generate KF.^[^ ^56^, ^57^, ^58^, ^59^ ^]^ Given that K⁺ exhibits the largest ionic radius (1.38 Å), the initially deposited products tend to form a loose, porous SEI structure due to poor ion transport. Consequently, electrolyte additives (e.g., adiponitrile, ADN) are required to induce product rearrangement. The cyano group (C≡N) in ADN can strongly adsorb and bind to K⁺, guiding the directional deposition of inorganic components such as KF and promoting SEI densification.^[^ ^60^ ^]^ In subsequent cycles, metallic potassium tends to form “dendrite‐dead potassium” complexes. The SEI must continuously replenish its KF layer via electrolyte decomposition to physically block dendrite penetration. Additionally, the outer nitrile polymer component can buffer volume expansion of the potassium electrode and inhibit side reactions induced by SEI cracking.

The charge density of Mg^2+^ is high, and its coordination with the electrolyte solvent is extremely strong, which significantly inhibits the solvent reduction reaction.^[^ ^61^, ^62^, ^63^, ^64^ ^]^ The SEI components are primarily derived from the decomposition of magnesium salts. During the initial charging process, common magnesium salts (e.g., magnesium bis(trifluoromethanesulfonyl)imide) undergo decomposition to form inorganic products such as MgF_2_ and Mg_3_N_2_.^[^ ^65^ ^]^ When a MgCl_2_‐based electrolyte is employed, a dense MgCl_2_ layer is formed. These inorganic products are directly deposited on the magnesium electrode surface, yielding a thin, dense initial SEI.^[^ ^66^ ^]^ However, the high charge of Mg^2^⁺ results in a higher migration barrier within the SEI, necessitating the regulation of SEI composition via boron‐based additives (e.g., trifluoroethoxyborane, tris(2,2,2‐trifluoroethyl)borate (TFEB)). TFEB can form coordination complexes with Mg^2^⁺; additionally, the introduction of a MgO‐B_2_O_3_ composite phase as an ionic transport channel reduces the migration resistance of Mg^2^⁺. Furthermore, the surface of metallic magnesium is prone to forming a native oxide layer (MgO/Mg(OH)_2_).^[^ ^22^ ^]^ This oxide layer exhibits poor ionic conductivity. The formation of the SEI thus requires that this oxide layer first be dissolved by anions such as Cl^−^ in the electrolyte, followed by the reconstruction of a stable, functional SEI to enable effective Mg^2^⁺ transport and electrode protection.

### 2.2 Microstructure Characteristics of SEI

SEI layer usually presents the characteristics of a multi‐layer structure.^[^ ^38^ ^]^ The inner layer near the anode surface often has a high content of inorganic components, and its structure is relatively dense, which mainly plays the role of stabilizing the electrode surface and preventing the electrolyte from further eroding the electrode. The outer layer contains more organic components, showing better flexibility and ion transport performance, which can facilitate the diffusion of Li^+^ in the SEI layer, and buffer the volume change of the electrode during the charging and discharging process. The design of this multilayer structure enables the SEI layer to achieve efficient ion transport while protecting the electrode. In addition, there are certain pore structures in SEI layer, which provide channels for the transmission of Li^+^. The size, distribution, and connectivity of pores have important effects on the transport rate of Li^+^. The proper pore structure can ensure the rapid and uniform migration of Li^+^ between the electrode and the electrolyte, and improve the charging and discharging efficiency of the battery.^[^ ^67^, ^68^ ^]^ However, if the pore structure is unreasonable, such as the pores being too small or blocked, it may lead to the blockage of Li^+^ transmission, increasing the internal resistance of the battery, and reducing the performance of the battery. Therefore, controlling the formation and evolution of pore structure during the formation of SEI layer is one of the keys to optimizing battery performance.^[^ ^41^ ^]^

### 2.3 Wetting Mechanism of SEI

Due to the presence of SEI, the wetting between the electrode and electrolyte is essentially the infiltration of SEI film and electrolyte. The wetting mechanism of SEI involves many aspects. From the point of view of surface energy, the electrolyte should overcome the surface tension between the SEI film and electrolyte to wet it. When the surface energy of the electrolyte matches with the surface energy of the SEI film, it is conducive to wetting.^[^ ^41^ ^]^ For example, if there are some polar groups on the surface of the SEI film, these groups can interact with the polar components in the electrolyte to reduce the surface tension, thereby promoting the spread of the electrolyte on the surface of the SEI film. In terms of microstructure, the pore structure of SEI film has an important effect on wetting.^[^ ^69^ ^]^ If the SEI film has the appropriate pore size and distribution, the electrolyte can enter these pores and fill them, just as water flows into the pores of sand. This pore‐filling process is an important part of wetting, enabling the electrolyte to contact the inside of the SEI film better, which in turn facilitates the transport of Li^+^ between the electrolyte and the SEI film.^[^ ^70^ ^]^

Filling is a wetting problem between the liquid electrolyte and porous electrode, which is essentially determined by the interaction between them. There is mutual attraction between liquid molecules, which causes the liquid surface to contract, thus forming surface tension, which plays a key role in the wetting process.^[^ ^71^ ^]^ For example, when the liquid is in contact with a solid surface, if the surface tension of the liquid is less than the critical surface tension of the solid surface, the liquid can spread out on the solid surface and achieve wetting. On the other hand, it is difficult to wet the solid surface, if the surface tension of the liquid is high. The angle formed between the tangent line of the liquid‐solid contact edge and the solid plane is defined as the contact angle. The smaller the contact angle, the higher the degree of wetting. When the contact angle is 0°, the liquid completely wets the solid. When the contact angle is between 0° and 90°, it is partially wetted. Greater than 90° is non‐wetting. In addition, factors such as the roughness of the solid surface, chemical composition, and the properties of the liquid have significant impacts on the wetting process. Rough surface will increase the actual contact area and enhance the wetting effect. In the ideal wetting case, where the solid surface is chemically uniform, atomically flat, and does not change during contact, the Neumann–Young equation (**Equation** **1**) shown below expresses the relationship between the contact angle and interface tension:^[^ ^71^ ^]^

(1) $$\gamma_{SV}=\gamma_{SL}+\gamma_{LV}\cos\theta$$

where *γ_SV_*, *γ_SL,_* and *γ_LV_* are solid‐vapor, solid‐liquid, and liquid‐vapor interfacial tension, respectively.

However, the actual surface of the porous electrode is very different from the ideal solid surface.^[^ ^72^ ^]^ The difference in physical microstructure or chemical topology of the active material particles will affect the contact angle of the electrolyte, and the interaction between the electrode surface and the liquid electrolyte molecules will lead to the difference in the wettability of different porous electrodes.^[^ ^15^, ^73^, ^74^ ^]^ In addition, external conditions such as temperature and voltage will also comprehensively act on the electrolyte wetting process, causing a change in contact angle.^[^ ^73^ ^]^

**Figure 3:**
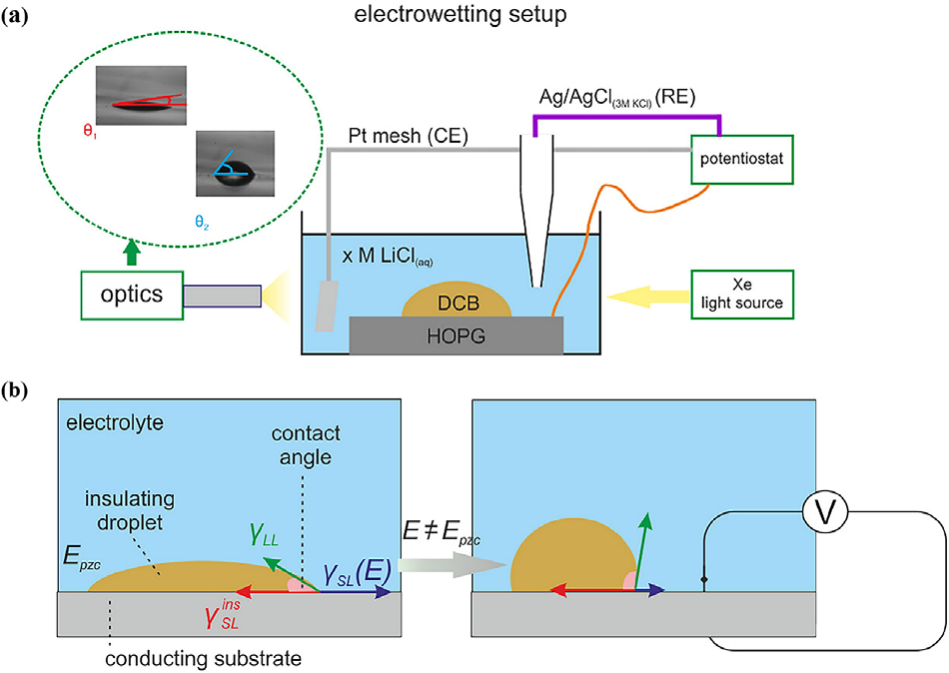
a) Schematic diagram of the experimental apparatus for electrowetting experiments. b) Schematic diagram of the electrowetting mechanism at the electrode/electrolyte/insulator interface.^[^ ^76^ ^]^ Copyright 2023, American Chemical Society.

In electrochemistry, the concept of electrowetting is also introduced recently. Electrowetting is a physical phenomenon, the electrocapillary phenomenon, originally discovered by Lippmann.^[^ ^75^ ^]^ Currently, the term electrowetting usually refers to the fact that the contact angle between the liquid and the solid surface changes when a voltage is applied to the liquid. In simple principle, the solid surface generally has a thin layer of dielectric, while the liquid maintains a certain contact angle on the solid surface when no voltage is applied. In the electrowetting device (**Figure** **3**a), when the insulated droplet stays at the zero charge potential (*E_pzc_*), where it exhibits the smallest contact angle. When a bias away from *E_pzc_* is applied, the reduction of *γ_SL_* (*E*) drives the receding motion of the insulated droplet, thereby increasing contact angle (Figure 3b). Electrowetting phenomena are usually described at the macroscopic level by the Young‐Lippmann equation (Equation 2), which predicts that the liquid‐solid contact angle (*θ*) decreases from its equilibrium value (*θ_0_*) at zero surface charge after voltage is applied. This depends on the magnitude of the applied voltage (*V*), the total area capacitance (*C_YL_*), and the liquid‐ambient (liquid‐air) surface tension (*γ_LV_*).^[^ ^76^ ^]^

(2) $$\cos\theta =\cos\theta_{0}+C_{YL}\frac{V^{2}}{2\gamma_{LV}}$$

Small additives have little effect on the properties of the electrolyte, while high concentrations of electrolytes or ionic liquid electrolytes may have an effect on the wetting mechanism. This is attributed to the movement of liquid in the capillary tubes, by the Washburn equation (**Equation** **3**)^[^ ^69^ ^]^:

(3) $$\frac{dh}{dt}=\frac{r^{2}}{8\eta h}\left(\frac{2\gamma_{LV}\cos\theta }{r}-\Delta \rho gh\right)$$

where *h* is the height of liquid penetration at time *t*, *r* is the radius of the capillary, *γ_LV_* is the surface tension between the liquid and the vapor, *θ* is the contact angle, *∆ρ* is the density difference, *η* is the viscosity.

### 2.4 The Development History of Philic‐Phobic Designs in Energy Storage Devices

The interface characteristics between electrodes and electrolytes exert a pivotal influence on the overall electrochemical performance of rechargeable batteries. The solid electrolyte interphase (SEI), a unique passivation film formed at the electrode/electrolyte interface, plays a decisive role in battery performance—its formation mechanism, microscopic structure, and intrinsic properties directly regulate key metrics including charge‐discharge efficiency, long‐term cycle stability, and operational safety of batteries.^[^ ^42^, ^43^, ^44^, ^45^ ^]^ With the ever‐increasing demand for high‐performance energy storage systems (particularly advanced batteries), gaining in‐depth insights into the SEI evolution mechanism in battery systems has become imperative.

**Figure 4:**
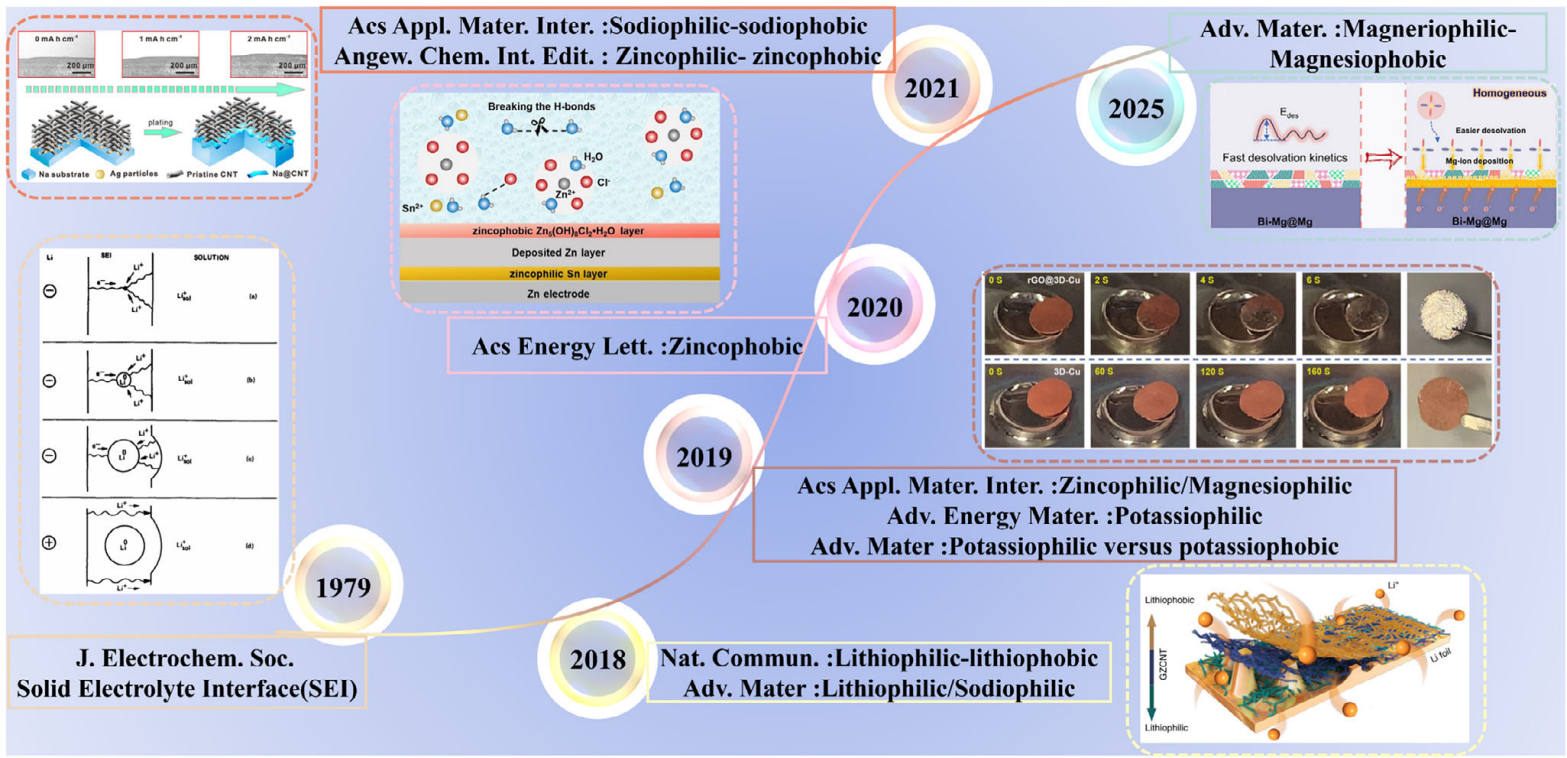
Milestone research on philic‐phobic designs in metal batteries. The first proposal of the SEI.^[^ ^77^ ^]^ Copyright 1979, IOP Publishing. Lithiophilic‐lithiophobic in lithium metal batteries.^[^ ^78^ ^]^ Copyright 2018, Springer Nature. Potassiophilic versus potassiophobic in potassium metal batteries.^[^ ^79^ ^]^ Copyright 2019, John Wiley and Sons. Zincophilic‐zincophobic in zinc metal battery.^[^ ^80^ ^]^ Copyright 2021, John Wiley and Sons. Sodiophilic‐sodiophobic in sodium metal battery.^[^ ^81^ ^]^ Copyright 2021, American Chemical Society. Magneriophilic‐magnesiophobic in magnesium metal battery.^[^ ^82^ ^]^ Copyright 2025, John Wiley and Sons.

The conceptual evolution of philic‐phobic designs in metal‐based batteries has experienced a progressive expansion, spanning from fundamental mechanistic discoveries to interface engineering in system‐specific scenarios (**Figure** **4**). In 1979, Peled pioneered the proposition of the SEI model, elucidating the critical function of a passivation layer endowed with ionic conductivity yet electronic insulation at the lithium metal/electrolyte interface.^[^ ^46^ ^]^ Subsequently, extensive research efforts have expanded this foundational framework to the rational design of customized interfaces leveraging “philic‐phobic” surface properties, marking a strategic shift from the general mechanistic understanding of SEI to metal‐specific interface modulation.^[^ ^47^, ^48^, ^49^, ^50^, ^51^, ^52^, ^53^, ^54^, ^55^, ^56^, ^57^, ^58^ ^]^ This advancement has laid a solid theoretical foundation for enhancing the long‐term cycling stability, operational safety, and energy density of next‐generation metal‐based batteries.

## 3 Superwetting Designs in Batteries

With the deepening of the study, superwetting no longer refers to the contact between the solid phase and liquid phase. In battery research, the infiltration of electrode and electrolyte essentially includes not only the infiltration of electrode‐electrolyte interface protective film, but also the contact between electrolyte and the SEI film, and even the contact between electrode and the SEI film. These different interfaces have great significant impacts on battery performances. Taking the Li metal battery as an example, during the first charging‐discharging process of the battery, the electrode and the electrolyte react to form a passivation layer covering the surface of the electrode material. This passivation layer is an interface layer, with the characteristics of a solid electrolyte, is an electronic insulator but a good conductor of Li^+^. The Li^+^ can be freely deposited and dissolved from the electrode across the passivation layer.^[^ ^83^, ^84^ ^]^ The SEI layer decisive impact on battery performance, and many studies are dedicated to designing multifunctional SEI layer, for example, the design of lithiophilic‐lithiophobic SEI in lithium metal battery and hydrophobic and zincophilic interfacial nanofilm in zinc metal battery.

### 3.1 Lithiophilic–Lithiophobic SEI

High voltage lithium metal batteries are regarded as an ideal candidate with great potential for future energy storage devices due to their high energy density and other advantages. However, the lithium metal anode exhibits the characteristics of thermodynamic instability and ultrahigh chemical activity.^[^ ^88^ ^]^ During the battery cycle tests, such instability and high activity can easily lead to the growth of lithium dendrites, and the production of dead lithium. The continuously growing lithium dendrites will puncture the separator, resulting in a short circuit of the cathode and anode electrodes. Dead lithium will cause the loss of active lithium, which in turn causes the CE of the battery to be greatly reduced, the lifespan of the battery will be significantly shortened, and in serious cases may even cause safety accidents such as explosions. The battery performances are hindered more seriously, especially in the case of high‐rate charging and discharging. This can be due to the unsatisfactory chemistry of the interface during the cycles and the poor transport dynamics of Li^+^. Between the lithium metal anode and the solid polymer electrolyte, the layered host structure with lithiophilic characteristics can play a notable role in inducing lithium to preferentially deposit in specific desired locations. In other words, this functional lithiophilic‐lithiophobic gradient SEI can effectively deal with the long‐term side reactions and lithium dendrite growth problems in the repeated lithium deposition or dissolution process of high‐energy‐density batteries based on solid polymers, thus providing a promising solution for improving the comprehensive performance of high‐voltage lithium metal batteries.

Therefore, the lithiophilic and lithiophobic SEI plays a significant role in many aspects in lithium metal batteries. First, the SEI can regulate the deposition behavior of Li^+^. The lithiophilic region can attract Li^+^ to preferentially deposit in specific locations, guide uniform nucleation and growth for lithium, and avoid the disordered growth of lithium dendrites. Lithium dendrites may puncture the separator and cause safety problems, while the construction of lithiophilic‐lithiophobic SEI can reduce this risk through regulated deposition, effectively improving the safety of the battery. Second, lithiophilic‐lithiophobic SEI helps to improve CE, and the lithiophobic region can reduce the side reactions between lithium and electrolyte, enabling more lithium to participate in the effective electrochemical reactions. Furthermore, lithiophilic‐lithiophobic SEI accelerates the Li^+^ transport dynamics, which can construct a channel or environment conducive to the migration of Li^+^, so that Li^+^ can be more smoothly and quickly transferred between cathode and anode electrodes during the battery charging and discharging process, which is particularly critical for the improvement of high‐rate performance.^[^ ^89^ ^]^ Thus, the comprehensive performance of the entire lithium metal battery is enhanced, laying a good foundation for practical application in energy storage and other fields. At present, it seems that the strategies of constructing lithiophilic‐lithiophobic SEI mainly relying on adding special additives to the electrolyte, material selection, and pretreatment, electrochemical regulation, and so on. A summary of the electrochemical properties of different electrolyte additives in lithium batteries is given in **Table** **1**. Compared with the electrolytes without additives, the addition of additives to construct lithiophilic‐lithiophobic SEI has obvious improvement on electrochemical performance.

**Table 1:** Comparison of electrochemical properties presented by different electrolyte additives in lithium batteries.

Types	Additive	Electrolyte	Ionic conductivity [mS cm^−1^]	Coulombic efficiency	Stability	Refs.
	SnF_2_	PEO	0.208	99%	71.3% 1000 cycles	[25]
Inorganic compound additive	No additive	PEO	0.144			
	Cu(NO_3_)_2_	FD	9.43	99.09%	93.1% 40 cycles	[85]
	No additive	FD	10.02	98.50%		
Organic compound additive	EVS+FEC	BE	10.70		97% 300 cycles	[18]
	No additive	BE	10.55		20% 300 cycles	
	AgTFSI	PEO	0.161		81.4% 500 cycles	[86]
Organic‐inorganic hybrid compounds	No additive	PEO	0.130		70.1% 500 cycles	
	FEC+LiNO_3_	LiTFSI‐DOL/DME		98.6%		[87]

In addition, artificial SEI is also a core solution for addressing the poor mechanical properties of native SEI, as well as the problems such as lithium dendrite growth and electrolyte decomposition. The precise design of hydrophobicity and hydrophilicity is the key strategy for enhancing the performance of artificial SEI. By regulating the hydrophilicity and hydrophobicity through molecular engineering or material composites, the artificial SEI can simultaneously achieve the dual functions of “lithium affinity” and “solvent repulsion”. An artificial SEI composed of polyethylenimine (PEI) and epoxysilane (EH) was constructed. Its lithium affinity can facilitate the uniform transmission and deposition of lithium ions and prevent the formation of dendrites. And with the increase in EH content, the enhanced solvent repellency can reduce the direct contact between the electrolyte and lithium metal, thereby lowering the probability of side reactions.^[^ ^34^ ^]^ Similarly, fluorine‐containing copolymer‐based artificial SEI achieves an improvement in interface stability through the ion conductivity of the inner inorganic material and the hydrophobic/hydrophilic nature of the outer polymer.^[^ ^90^ ^]^

#### 3.1.1 Inorganic Additives

First of all, the addition of inorganic additives can regulate the deposition process of Li^+^, which can be preferentially adsorbed on the surface of lithium metal. For example, some fluorine‐containing inorganic additives, such as LiF, can be adsorbed on the surface of lithium, increasing the initial sites of lithium deposition. These sites are helpful to regulate Li^+^ to deposit evenly, just like pouring liquid on a template with many small pits, the liquid (Li^+^) will be more evenly distributed in these small pits (adsorption sites), thus inhibiting the growth of lithium dendrites and changing the nucleation overpotential of lithium deposition. Additives like some inorganic borates can reduce the nucleation overpotential of lithium deposits, which means that Li^+^ is easier to start deposition on the electrode surface, and the deposition process is more stable, which is conducive to the formation of a dense and flat lithium deposit layer, and then affects the lithiophilic and lithiophobic properties of the SEI layer.

Second, inorganic additives can also react with lithium salts and solvents in the electrolyte during the first charging and discharging of the battery. For instance, LiNO_3_ can react with lithium metal on the electrode surface to form an SEI layer containing Li_2_O, Li_3_N, and other components. Among them, components such as Li_2_O can provide lithiophilic sites, which are conducive to the transfer of Li^+^. The components, such as Li_3_N can regulate the structure of the SEI layer to make it lithiophobic to a certain extent to prevent the excessive growth of lithium dendrites. These reactions generate inorganic compounds that can adjust the composition and structure of the SEI layer. Besides, the composition of the SEI layer formed by the inorganic carbonate after the reaction can increase the mechanical strength of the SEI layer, making the SEI layer less likely to break during the repeated deposition/stripping of lithium metal. The inorganic carbonate can adjust the transmission path of Li^+^ in the SEI layer via constructing lithiophilic–lithiophobic gradient structure.

In addition, inorganic compound additives can also affect the Li^+^ transport behavior and change the Li^+^ conductivity of the SEI layer. For example, some inorganic oxides with high Li^+^ conductivity, such as Li_4_Ti_5_O_12_, increase the rate of Li^+^ conduction in the SEI layer when it participates as an additive. In this way, during the battery charging and discharging process, Li^+^ can pass through the SEI layer more smoothly. According to inorganic oxides with high Li^+^ conductivity distribution and structure in the SEI layer, a lithium‐friendly transmission channel is formed on the side near the lithium metal, and a lithium‐phobic barrier layer is formed on the side away from the lithium metal to prevent the lithium dendrites from piercing the SEI layer and adjust the diffusion path of Li^+^. Certain inorganic halide additives can form nanoscale channels or lattice defects in the SEI layer, which can guide Li^+^ to spread along a specific path, as if the correct route is set in a maze. This adjustment of the Li^+^ diffusion path helps to construct a lithiophilic‐lithiophobic SEI layer, so that Li^+^ can preferentially transport in the lithiophilic region, while the lithium phobic region can play a protective role.

**Figure 5:**
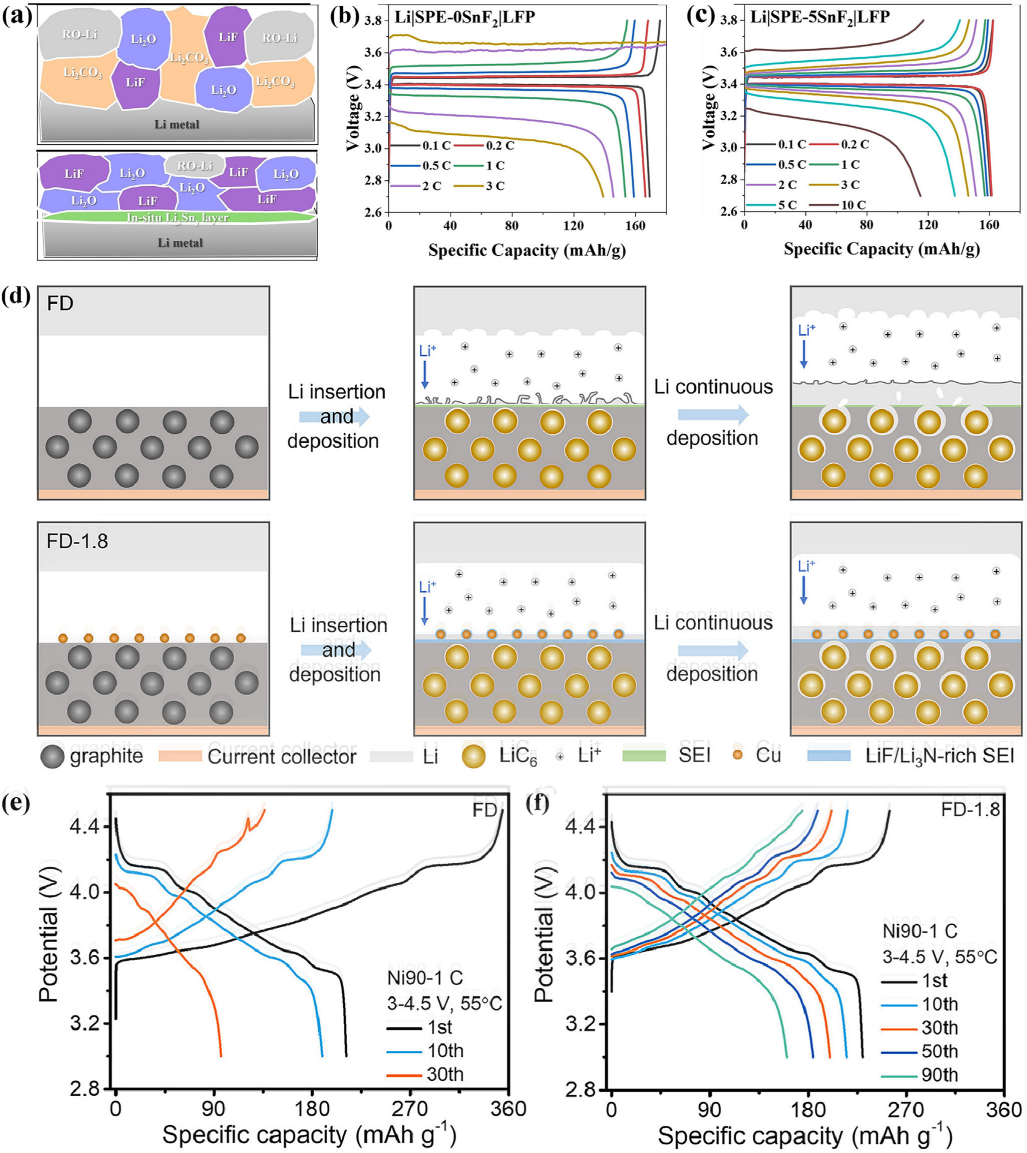
a) Schematic illustration of SEI components at the surface of cycled Li metal anode based on SPE‐0SnF_2_ and SPE‐5SnF_2_. Charging‐discharging voltage profiles of b) Li|SPE‐0SnF_2_|LFP and c) Li|SPE‐5SnF_2_|LFP at different rates at 60 °C.^[^ ^25^ ^]^ Copyright 2024, John Wiley and Sons. d) Schematic illustration of deposition process of lithium on graphite electrode in different electrolytes. Capacity voltage profiles of e) FD and (f) FD‐1.8 in the specific cycles.^[^ ^85^ ^]^ Copyright 2024, Elsevier.

All‐solid lithium metal batteries have excellent safety, with solid polymer electrolytes (SPE) that are not easy to leak and have excellent mechanical properties. Among them, polyethylene oxide (PEO) based electrolyte has attracted much attention and became a research hotspot in the SPE research field due to its excellent mechanical properties, outstanding processability and positive compatibility with electrodes. However, pure PEO electrolyte is not perfect, and there are always insurmountable side reactions and lithium dendrite growth in the practical application process, which are particularly serious under high current conditions, everely restricting the further development and application of pure PEO electrolyte. To solve this problem, a lithiophilic‐lithiophobic gradient interface composed of Li_x_Sn_y_/LiF‐Li_2_O was successfully constructed through the catalytic of tin difluoride (SnF_2_), which dramatically enhanced the rate performance of PEO‐based all‐solid‐state batteries (**Figure** **5**a). In this interface, the upper layer is rich in ionic states of LiF‐Li_2_O (17.1 nm thick layer), which not only has high interface energy, but also provides rapid Li^+^ diffusion channels. Specifically, the discharging capacity of the Li|SPE‐0SnF_2_|LFP battery gradually decreases at 3C, or even drops to zero (Figure 5b). The discharging capacity of the Li|SPE‐5SnF_2_|LFP battery can recover back to 162 mAh g^−1^ when the rate is returned to 0.1C, which indicates that SPE‐5SnF_2_ has good stability and further promotes the stable transmission of Li^+^ (Figure 5c). At the same time, the underlying Li_x_Sn_y_ alloy (8.4 nm thick layer) effectively reduces the nucleation overpotential and the resistance to Li^+^ diffusion and promotes the rapid transfer of electrons, thus ensuring the reversible deposition and release of Li^+^. In addition, insoluble SnF_2_ works with PEO further increase the rate of transport of Li^+^ ions in the bulk phase. Thanks to this innovative interface design, the cycle life and critical current density of the symmetric battery have been increased by up to 46.7 times and 3.5 times, respectively.^[^ ^25^ ^]^ This reaffirms the importance of the lithiophilic‐lithiophobic gradient interface in the design of high rate all‐solid‐state lithium metal batteries that can be safely applied.

The chemical composition and microstructure of SEI occupy a central position in the determining mechanism of physicochemical properties, and have a fundamental impact on the electrochemical behavior and stability of the battery system. In many types of SEI, inorganic SEI is widely recognized as a favorable variant of SEI because it is rich in lithium‐phobic inorganic components such as LiF and Li_3_N. From the perspective of electrochemical kinetics, the special composition significantly promotes the formation of a uniform and continuous SEI layer on the electrode surface, which apparently inhibits the growth of lithium dendrites and excessive decomposition of electrolytes. In terms of mechanical properties, SEI can withstand large stress and strain during the charging and discharging cycles, and maintain the stability of the electrode‐electrolyte interface because of excellent mechanical robustness. The construction of SEI significantly reduces the fluctuation of the battery internal resistance and improves the cycle life and safety performance threshold, providing a reliable solution for the construction of high‐performance battery systems.

In addition, FD electrolyte is introduced, which consists of 1 M LiPF_6_ in fluoroethylene carbonate (FEC) and dimethyl carbonate (volume ratio 3:7).^[^ ^85^ ^]^ When 1.8%, Cu(NO_3_)_2_ of the total mass of the solvent is added, the electrolyte is named FD‐1.8 respectively. Cu(NO_3_)_2_ can not only promote the diffusion of Li thus decreasing the Li concentration gradient, but also be reduced on the anode surface to form the lithiophobic Cu particles and propel lithium to realize dense deposition. During this process, FEC and NO^3^ ^−^ anion simultaneously participate in the formation of SEI, and thus a LiF‐enriched Li_3_N lithiophilic layer was built (Figure 5d). Comparing FD‐based batteries with FD‐1.8 batteries, batteries using FD‐1.8 exhibit higher CE, better cycle stability within 90 cycles, and less polarization change (Figure 5e,f). It is further verified that Cu(NO_3_)_2_ also plays a crucial role in cathodic protection. Therefore, layered lithiophilic and lithiophobic bodies can be in situ formed through simple electrolyte engineering. The concept has been tested on a variety of anodes, including the metals lithium, copper, and graphite.^[^ ^85^, ^91^, ^92^ ^]^

#### 3.1.2 Organic Additives

In addition to inorganic additives, organic additives also play a significant role that cannot be underestimated. When the battery begins to charge, the organic compound electrolyte additive will undergo a reductive decomposition reaction on the electrode surface, which due to the additive molecule can gain electrons at negative potential. For example, carbonate additives (such as vinyl carbonate) get electrons under low voltage, and the following ring‐opening reactions occur to produce products such as lithium alkyl carbonate.^[^ ^93^, ^94^ ^]^ These decomposition products are deposited on the electrode surface and become the initial components of the SEI film. A similar situation applies to organic sulfide additives, like dimethyl sulfoxide, its reduction on the electrode surface produces sulfur‐containing compounds that participate in the construction of SEI films.^[^ ^95^, ^96^ ^]^ From the perspective of molecular orbital theory, the lowest unoccupied orbital level of these additive molecules can accept electrons at the reduction potential of the electrode, thus triggering the decomposition reaction. The decomposition products interact and form the basic skeleton structure of SEI. For the formation of lithiophilic SEI, some decomposition products containing lithiophilic functional groups (such as carbonyl groups, ether bonds, etc.) tend to be close to the lithium electrode side. These functional groups can interact with Li^+^ through coordination and other forces, so that Li^+^ gather around them. From the perspective of crystal structure, part of the decomposition products will form inorganic substances with a certain lattice structure (such as lithium salt crystals), while other parts of the organic components are filled in the gaps of inorganic substances, jointly building the SEI structure that is conducive to ion migration and can block the conduction of electrons. Taking fluorine‐containing organic additives as an example, the LiF produced by their decomposition can form a stable inorganic phase.^[^ ^97^ ^]^ The LiF is intertwined with other consequential organic components, which plays an inhibitory role in the growth of lithium dendrites.

In addition, the SEI film formed by the organic compound electrolyte additive can regulate the transport channel of Li^+^, where the lithiophilic region can make it easier for Li^+^ to adsorb and detach, which is similar to providing an active site during ion exchange. The lithiophilic design reduces the activation energy of Li^+^ transport, making the diffusion coefficient of Li^+^ relatively high in this region. The lithiophobic region can prevent the excessive growth of lithium dendrites. For instance, some additives with long‐chain alkyl structure decomposition products will form a lithiophobic layer, hindering the disordered growth of lithium dendrites due to the steric hindrance and hydrophobicity of the alkyl chain.^[^ ^98^ ^]^ Thus, Li^+^ in this region can still be transferred through specific channels (such as through gaps or defects in organic components), but the transmission rate is lower than that in lithiophilic region. The organic compound electrolyte additives contribute to stabilize the electrode/electrolyte interface during the battery cycles. As a buffer layer, SEI film avoids direct contact between electrode and electrolyte, reducing the occurrence of side reactions. The SEI film with lithiophilic and lithiophobic structure can better adapt to the volume changes of Li^+^ during the process of depositing and dissolving. The stable lithiophilic and lithiophobic SEI film can keep the interface charge transfer impedance at a low level, which is conducive to the charging and discharging efficiency and cycle life of the battery. The forming SEI film can maintain a low interface impedance under fast charging and discharging conditions by adding organic additives containing boron, dramatically protecting the electrode surface.^[^ ^97^, ^99^ ^]^

**Figure 6:**
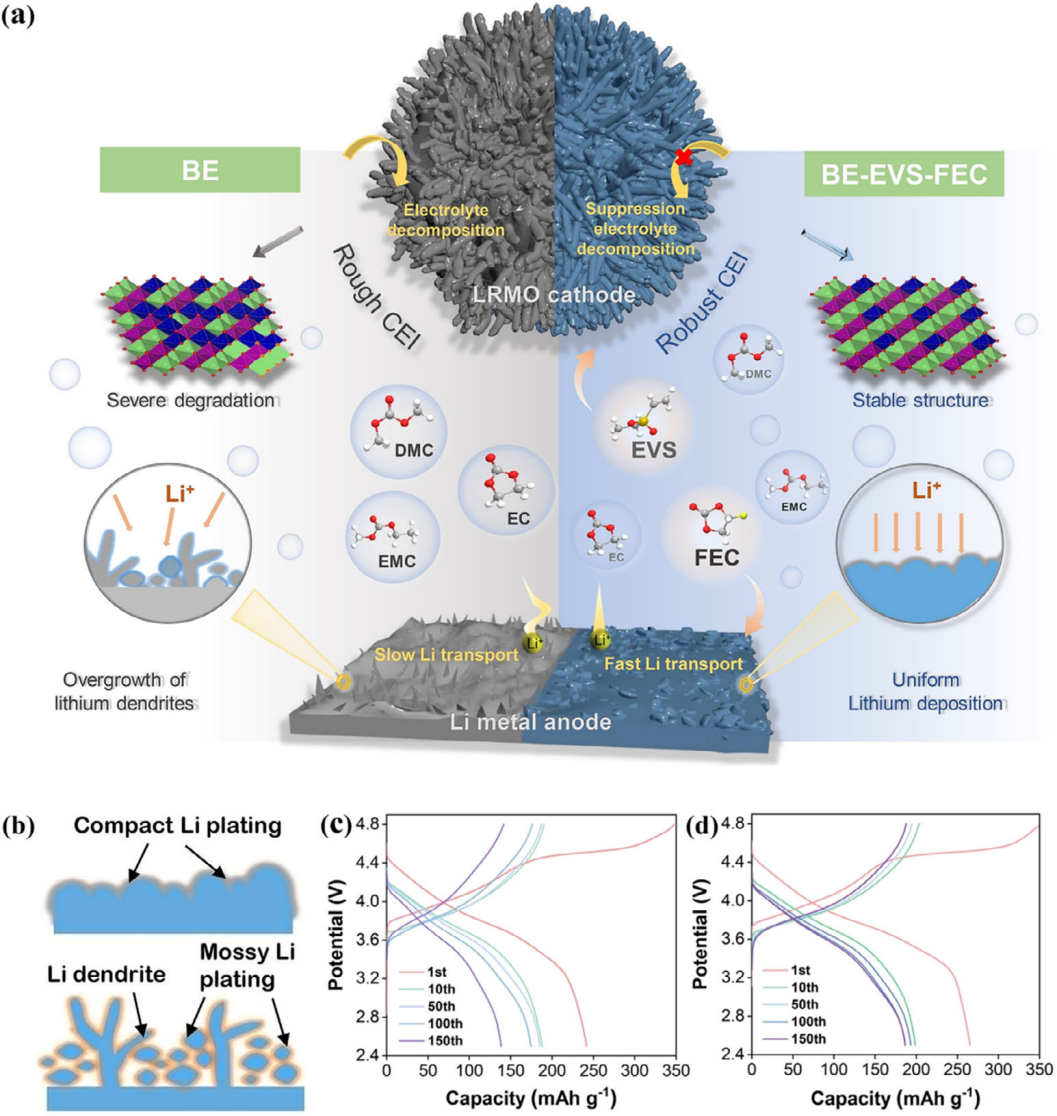
a) Working mechanism of as‐proposed dual‐additive formulation on regulating the electrode/electrolyte interfaces in LRMO|Li battery system. b)Schematic representation of the deposition of samples cycled with BE and BE‐EVS‐FEC electrolytes on the surface of lithium metal. Charging‐discharging curves LRMO|Li with c) BE and d) BE‐EVS‐FEC electrolytes.^[^ ^18^ ^]^ Copyright 2023, John Wiley and Sons.

Moreover, the high‐voltage lithium‐rich manganese oxide (LRMO) cathode was studied. The baseline electrolyte (BE) was 1 M LiPF_6_ in EC/EMC/DMC solution (1:1:1, by mass). Ethylene ethyl sulfone (EVS) and FEC were also innovatively proposed using as composite additives to construct an electrolyte with affinity for both high‐voltage positive and highly active lithium negative electrodes. Such a strategy successfully overcomes the difficulty that the traditional electrolyte can not be compatible with the high voltage cathode and the lithium metal cathode at the same time (**Figure** **6**).^[^ ^18^ ^]^

#### 3.1.3 Organic–Inorganic Hybrid Additives

SEI formed by some organic‐inorganic hybrid electrolyte additives exhibit lithiophilic properties. These SEI may contain functional groups that can chemically adsorb or coordinate with Li^+^, such as carbonyl groups (C═O) and hydroxyl groups (‐OH).^[^ ^100^ ^]^ The lone pair electrons of these functional groups can form coordination bonds with Li^+^, allowing Li^+^ to enrich in their vicinity. For example, some organic‐inorganic hybrid additives containing carboxyl groups, after reduction and decomposition, carboxyl groups will combine with Li^+^ to form a lithiophilic region, which is conducive to the deposition of Li^+^.^[^ ^2^, ^86^, ^87^ ^]^ Some other components produced by the decomposition of hybrid additives simultaneously show lithiophobic properties. Some inorganic‐organic compounds containing long‐chain alkyl or silyl groups will spread out to form a lithiophobic layer after decomposition. These lithiophobic components can prevent the excessively grown lithium dendrites from penetrating the SEI film and contacting the electrolyte directly.

Moreover, the lithiophobic component can regulate the transport path of Li^+^, so that the Li^+^ can be orderly transported between the lithiophobic and lithiophobic regions. The special multi‐level SEI structure was constructed by interweaving lithiophilic and lithiophobic components.^[^ ^78^ ^]^ The lithiophilic region may be closer to the electrode surface, providing a rapid deposition channel for Li^+^.^[^ ^101^, ^102^ ^]^ Correspondingly, the lithium‐phobic region is on the outside, which plays a role in protecting and regulating the transmission of Li^+^. Such a multilayer structure, similar to a functionally graded material, is better suited to adapt to the volume changes during the deposition and dissolution of Li^+^. During the battery cycles, the SEI film constructed by the organic‐inorganic hybrid electrolyte additive is constantly affected by the Li^+^ deposition/dissolution processes. The lithiophilic‐lithiophobic structure could maintain a dynamic equilibrium in these repeated charging and discharging process. Specifically, when Li^+^ deposited in large numbers, the lithiophilic region may expand to a certain extent, and the lithiophobic region will adjust structure accordingly to accommodate this change. The various components in the SEI film will maintain the stability of their functional structure through ion exchange, fracture and formation of chemical bonds guided by chemical balance. If the SEI film is locally damaged during the battery cycles, the organic‐inorganic hybrid compound electrolyte additive can self‐repair through its own decomposition or reaction with surrounding components.^[^ ^2^ ^]^ When cracks appear in the lithium philic region of the SEI film, some components in the additive may undergo a reduction reaction at the crack, re‐forming the lithium philic component, filling the crack, restoring the integrity of the SEI film, and thus extending the service lifespan of the battery.^[^ ^103^ ^]^

**Figure 7:**
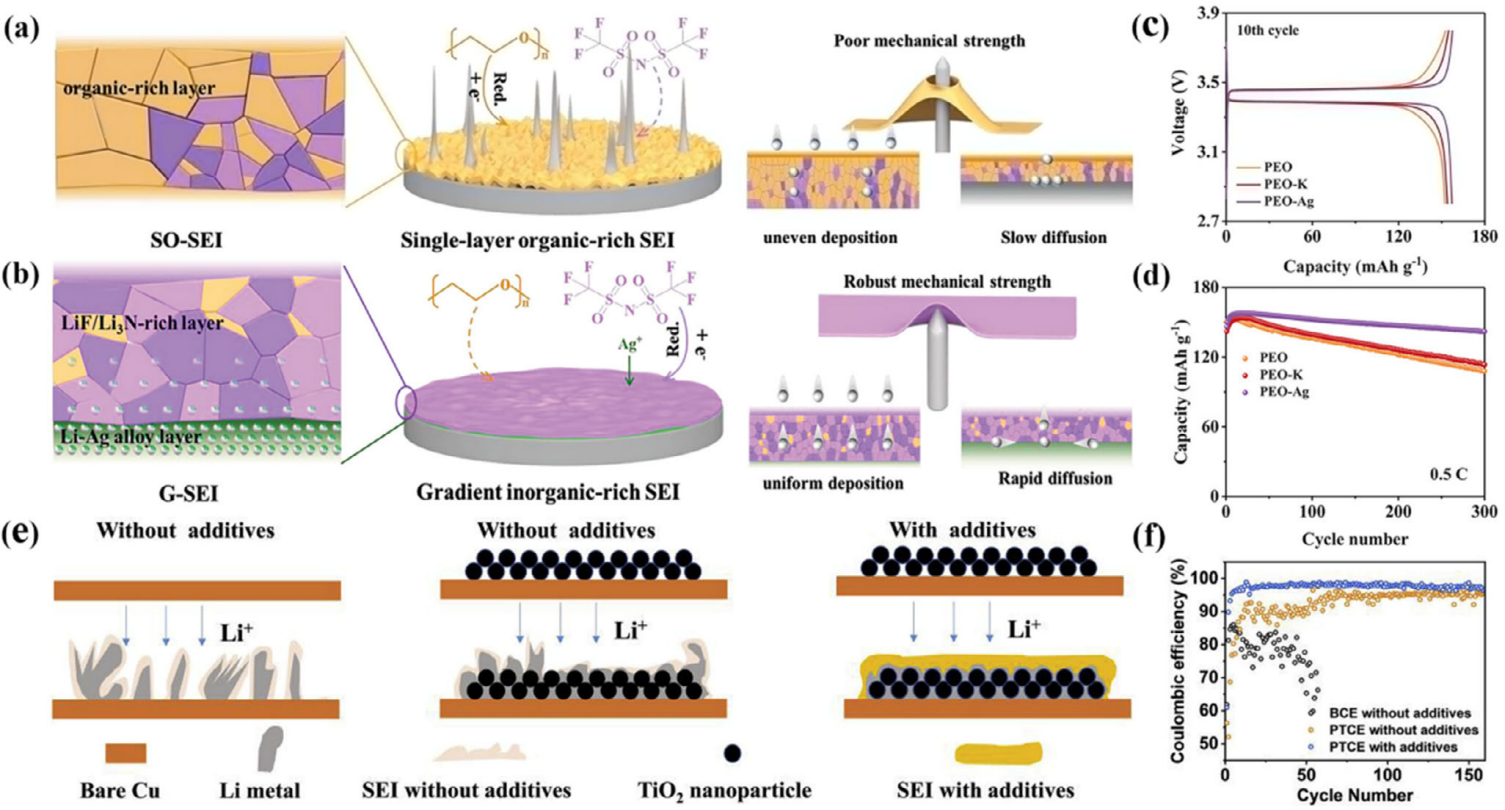
The illustration of the structural evolution of a) common single‐layer organic‐rich SEI (SO‐SEI) and b) dual‐layer inorganic‐rich (G‐SEI) during Li plating. c) Charging‐discharging curves of Li‐LFP cells at 0.5C after 10 cycles. d) Cycling performances of the Li‐LFP cells at 0.5C.^[^ ^86^ ^]^ Copyright 2024, John Wiley and Sons. e) Schematic diagram of lithium plating on additive free BCE, additive free or additive containing PTCE. f) Cycling performance of these three styles of symmetric cells.^[^ ^87^ ^]^ Copyright 2019, Elsevier.

Besides, due to the low mechanical stability of the SEI, the stress distribution within the SEI becomes uneven during uneven lithium deposition/stripping, which causes the SEI to constantly break and regenerate (**Figure** **7**a). AgTFSI was also used as an additive in polyoxyethylene electrolyte (PEO‐Ag) to in situ construct stable LiAg‐LiF/Li_3_N lithiophilic‐lithiophobic gradient SEI (G‐SEI) (Figure 7b).^[^ ^86^ ^]^ In the upper LiF/Li_3_N Li‐rich layer, the LiF interface energy is high, and the Li^+^ diffusion of Li_3_N is rapid, and they naturally cooperate to promote the uniform deposition of Li^+^. The lithiophilic Li‐Ag alloy can reduce nucleation overpotential and promote the growth of lithium plane. The G‐SEI simultaneously has a high mechanical modulus, which can prevent dendrites from penetrating SEI and avoid continuous degradation of PEO based electrolyte.^[^ ^86^ ^]^ At a low current density of 0.5 C, Li|PEO|LFP, Li|PEO‐K|LFP, and Li|PEO‐Ag|LFP provide initial discharge capacities of 152.5, 153.8, and 157.2 mAh g^−1^, respectively. After 300 cycles, the Li‐LFP battery with the PEO‐Ag electrolyte maintained the highest capacity, much higher than the other two batteries (Figure 7c,d). In short, the research and development of lithiophilic‐lithiophobic gradient SEI has opened up a considerable path for the realization of high‐performance solid‐state lithium batteries. Porous TiO_2_ scaffolds were also developed to regulate lithium nucleation and inhibit lithium dendrite growth.^[^ ^87^ ^]^ The lithiated stent can alleviate the volume change during lithium plating/stripping, which dramatically reduce the local current density and inhibits the lithium dendrites. A LiF‐rich SEI layer formed on the surface of porous TiO_2_ modified Cu electrode (PTCE) after the FEC and LiNO_3_ were added to the ether‐based electrolyte, which effectively prevents the decomposition of the electrolyte and the further growth of lithium dendrites (Figure 7e).^[^ ^87^ ^]^ PTCE samples with additives and without additives showed long‐term stability up to 900 h (225 cycles) and 700 h (175 cycles), respectively, while the bare Cu electrode (BCE) samples without additives began to oscillate at ≈200 h (50 cycles) (Figure 7f). It is shown that the SEI layer generated by introducing additives and constructing porous TiO_2_ scaffold can effectively inhibit the growth of lithium dendrites and the endless decomposition of electrolyte.

### 3.2 Zincophilic–Zincophobic SEI

Zinc metal battery is an outstanding representative of promising aqueous electrolyte‐based batteries. In the field of zinc metal batteries, the construction of zincophilic and zincophobic double‐layer interface has great meanings to improve battery performances recently.^[^ ^104^ ^]^ In the battery system, when the zinc metal anode is in contact with the electrolyte, a series of electrochemical reactions and interface processes will occur immediately.^[^ ^105^, ^106^, ^107^ ^]^ For example, some additives or solvent molecules with specific functional groups may pick up electrons on the surface of zinc anode, forming an adsorbed layer containing organic components.^[^ ^2^, ^108^, ^109^ ^]^ The polar functional groups in these organic components, such as carbonyl, amino, and so on, can interact with zinc ions to a certain extent, thus showing the zincophilic properties, and they finally become a significant part of the zincophilic layer. The formation of this zincophilic layer is conducive to the uniform deposition of zinc ions on the electrode surface, which can guide the subsequent deposition behavior of zinc and weaken the formation tendency of zinc dendrites. Anions or other inorganic components in the electrolyte, meanwhile, will also participate in the interface reactions. For instance, sulfate ions may react with zinc ions to form some inorganic salts of zinc, such as zinc sulfate deposits.^[^ ^110^ ^]^ Due to their crystal structure and chemical bonding characteristics, the affinity of these inorganic salts to zinc is relatively weak, showing zinc repellency. With the progress of the reaction, this part of the zincophobic inorganic salt gradually deposited and grew on the zincophilic layer, forming a zincophobic layer, which can prevent the further growth and penetration of zinc dendrites as well as the damage of dendrites to the separator and the resulting short circuit of the battery.

During the charging‐discharging cycle of the battery, the zincophilic and zincophobic double‐phase interface is constantly dynamically adjusted and optimized. Zinc ions dissolve from the electrode surface into the electrolyte under charging, and the organic components in the zincophilic layer may undergo a certain degree of structural rearrangement or dissociation‐recombination process with zinc ions to adapt to the change in zinc ion concentration. The crystal structure of the inorganic salt in the zincophobic layer may also undergo a small crystal transformation or defect repair due to the change of ion concentration and electric field, so as to maintain resistance to zinc dendrites. Zinc ions are redeposited on the electrode surface under the discharging process, and the zincophilic layer can effectively guide the uniform nucleation and growth of zinc ions on the surface of Zn anode, making the zinc deposited layer flatter and denser. The zincophobic layer continues to play its shielding role, preventing zinc dendrites from breaking through the interface layer.^[^ ^113^ ^]^ Besides, external factors such as temperature and current density also affect the formation and stability of the zincophilic‐zincophilic double‐layer interface. The higher temperature may accelerate the reaction rate of electrolyte components on the surface of the zinc electrode and promote the rapid formation of the interface layer, nevertheless, it may lead to the increase of the non‐uniformity of the interface layer. However, excessive current density may cause the deposition rate of zinc ions to be too fast, which exceeds the guiding and regulating ability of the zincophilic layer, thus destroying the structural integrity of the double‐layer interface. By adjusting these external factors reasonably, the performances of the zincophilic and zincophobic double‐phase interface can be further optimized, and the key performance indexes such as cycle life, CE, and safety of zinc metal batteries can be improved.^[^ ^114^ ^]^ The electrochemical properties of zinc metal batteries for including different additives to electrolytes are summarized and compared in **Table** **2**. Compared with the contact angle between the SEI and the electrolyte without additives, the contact angle generated by adding additives is obviously decreased, which is conducive to the ion transport and battery safety.

**Table 2:** Comparison of electrochemical properties and contact angles of different electrolytes in zinc batteries.

Additive	Electrolyte	Contact angle	With additive contact angle	Coulombic efficiency	Stability	Refs.
SnCl_2_	ZnCl_2_	137°		99.9%	95% 200 cycles	[80]
InCl_3_+EG	Zn(ClO_4_)_2_	38.1°	54.3°	99.9%	93.1% 2000 cycles	[111]
SN	ZnSO_4_	15°	22°	99.7%	3.5% 50 cycles	[19]
AS	ZnSO_4_, Zn(OTF)_4_	67.38°	76.98°	99.6%	95.92% 3000 cycles	[112]

#### 3.2.1 Eutectic Electrolyte System

**Figure 8:**
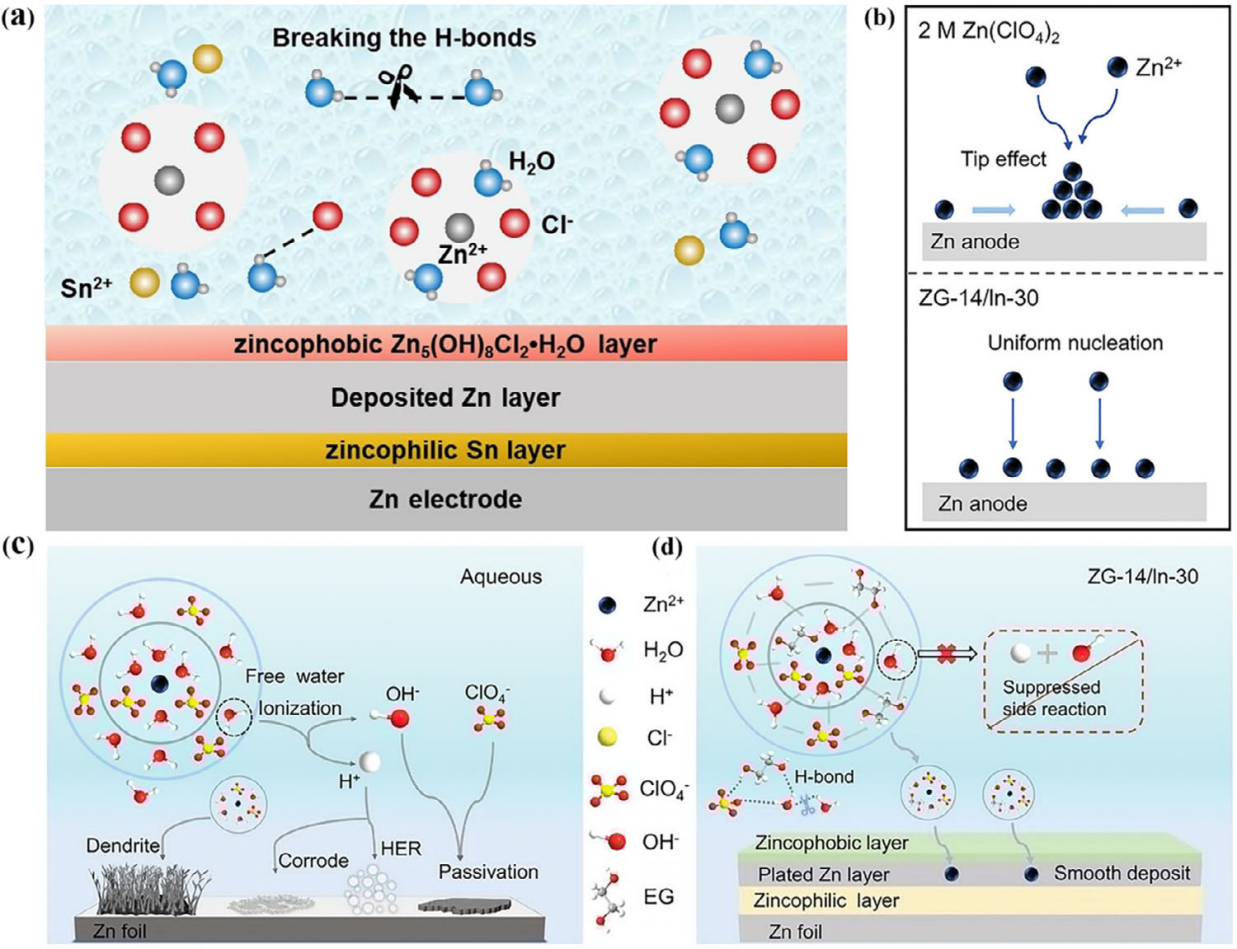
a)Scheme of electrolyte and electrolyte–electrode‐interphase structure.^[^ ^80^ ^]^ Copyright 2021, John Wiley and Sons. b) Schematic diagram of Zn^2+^ diffusion and nucleation of Zn surfaces in different electrolytes. Schematic diagrams of the Zn^2+^ solvation shell and electrochemistry behaviors at the Zn/electrolyte interface in c) an aqueous system and d) a hybrid hydrated eutectic system.^[^ ^111^ ^]^ Copyright 2023, John Wiley and Sons.

The cycle life of zinc batteries is challenged by low CE, water consumption, and growth of zinc dendrites, which seriously restrict the large‐scale development of zinc batteries. Poor low‐temperature performances also limit the practical applications of zinc batteries. Recent research advances have focused on design strategies at the molecular level, successfully reducing the water electrolyte solidification temperature to ‐70 °C,^[^ ^80^ ^]^ and further building the SEI to inhibit zinc dendrite runaway formation and improve of CE. Nevertheless, the mechanism of how the physicochemical properties of the electrolyte affect the formation of SEI film on the negative zinc anode surface is still unclear and needs further exploration. To clarify this issue, Wang et al. put to use eutectic 7.6 m ZnCl_2_ aqueous solution and 0.05 m SnCl_2_ solution to effectively overcome the two problems of unrestricted growth of zinc dendrites and poor performance at low temperature.^[^ ^80^ ^]^ In this process, the electrolyte in situ forms a zincophilic‐zincophobic Sn/Zn_5_(OH)_8_Cl_2_•H_2_O dual‐layer interface phase (**Figure** **8**a), resulting in high cycling stability at low temperature. Further studies show that zincophilic tin can reduce zinc deposition/stripping potential and promote uniform deposition of zinc ion. In addition, the eutectic electrolyte exhibit a high ionic conductivity of 0.8 mS cm^−1^ even at a low temperature of −70 °C due to the deformation of the hydrogen bond network caused by solvation of Zn^2+^. Besides, recently, Wan et al. designed a hydrated eutectic electrolyte system consisting of Zn(ClO_4_)_2_·6H_2_O, ethylene glycol (EG), and InCl_3_ additives.^[^ ^111^ ^]^ The electrolyte enables aqueous zinc‐ion batteries to operate over a wide temperature ranging from −50 to 50 °C. As shown in Figure 8b, Zn^2+^ diffuses to the tips, gradually promoting the growth of Zn dendrites in different electrolytes. During battery operation, these complex molecules in the hydrated eutectic electrolyte break down, resulting in the formation of a double‐layer interface consisting of a zincophobic‐zincophilic layer on the zinc anode surface (Figure 8c,d). Specifically, the zincophilic interface reduces the energy barrier of zinc nucleation and induces uniform deposition of zinc ion. On the other hand, the zinc‐phobic interface also acts as a barrier to prevent water from penetrating the surface of zinc anode and other side reactions. Therefore, these hydrated eutectic electrolytes contribute to the thermodynamic stability of the zinc negative electrode and inhibits the growth of zinc dendrites and guide the uniform deposition of zinc ion.

#### 3.2.2 Electrolyte Additives

**Figure 9:**
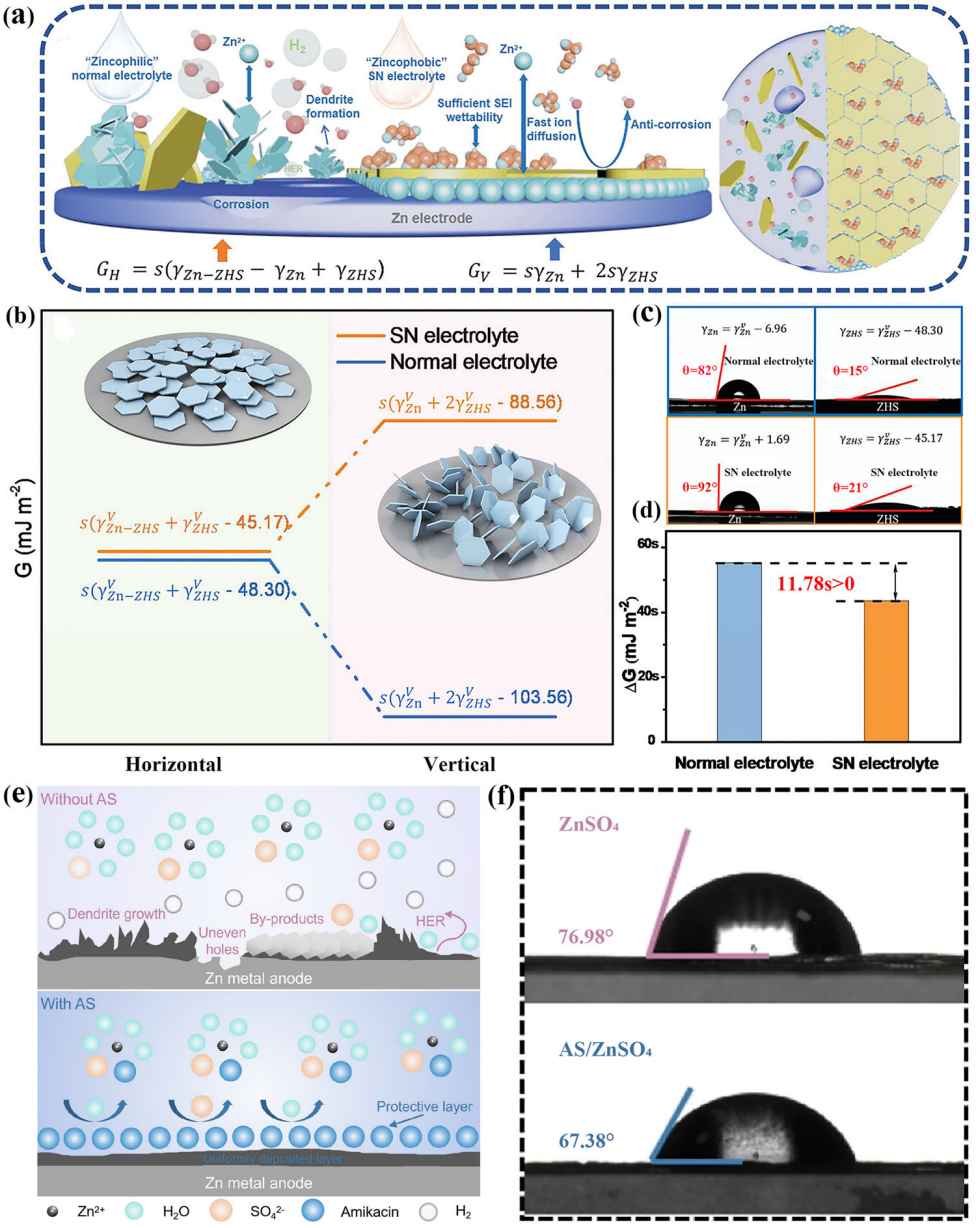
a) Schematic of zincophobic but SEI‐affinity electrolyte. b) Calculation expressions of free energy and free energy in two growth states in SN electrolyte and normal electrolyte. c) Contact angle test and corresponding detailed surface energy. d) Comparison of the amount of change in free energy in two electrolytes.^[^ ^19^ ^]^ Copyright 2023, John Wiley and Sons. e) Schematic illustrations of Zn deposition with/without AS. f) The contact angles of the electrolyte with/without AS on the Zn surface.^[^ ^112^ ^]^ Copyright 2025, Elsevier.

From a practical application point of view, a small amount of additives can greatly optimize the electrolyte environment and improve the stability of the interface between electrode and electrolyte. In zinc batteries, succinonitrile (SN), as a typical additive in the electrolyte, can be reduced and decomposed under zinc anode potential recently.^[^ ^2^, ^19^, ^115^ ^]^ The zincophilic interface formed by the interaction of sulfur groups with zinc ions can reduce the deposition energy barrier of zinc and inhibit dendrite growth. The nitrogenous substances produced by decomposition build a zinc‐phobic network, which allows zinc ions to pass through properly under the action of electric field, but can prevent solvent molecules and impurities from migrating to the zinc anode, meaning fewer side reactions. During long‐term cycles, SN can repair micro‐cracks or defects in SEI layer caused by changes in zinc volume, maintain stable zincophilic and zincophobic functions, and ensure excellent cycle performance and electrochemical stability. Wang et al. innovatively developed a zinc‐phobic electrolyte with SN additive, exhibiting unique affinity for zinc, but has strong binding force with SEI.^[^ ^19^ ^]^ In the condition of SN electrolyte, ZHS will preferentially accumulate horizontally, and then construct a dense SEI protective layer on the surface of Zn anode, which effectively cut off the corrosion of Zn and the breeding of dendrites. In **Figure** **9**a–d, a schematic of the SEI is shown, along with a comparison of the contact angle and free energy with/without electrolyte additives. This zinc‐phobic SN electrolyte exhibits excellent performance, and a high CE of 99.71% was achieved after 400 galvanizing/stripping cycles. In the symmetric battery, when the zinc utilization rate is 0.9%, it can be stable for 4000 h, and when the zinc utilization rate is increased to 86.1%, it can still be stable for 325 h.^[^ ^19^ ^]^

In addition, the additive strategies for zincophilic groups have received extensive attention. Zincophilic functional groups generally have chemical structures that attract zinc ions to each other. For instance, some sulfur‐containing functional groups (e.g., ‐SH, ‐S‐S‐) can interact with zinc ions through chemical bonds (e.g., ionic bonds, coordination bonds).^[^ ^116^ ^]^ In the zinc ion deposition stage, these zincophilic functional groups can provide preferential deposition sites. They adsorb zinc ions, causing zinc ions to accumulate near them, promoting zinc deposition in a more uniform manner, and inhibiting the formation of zinc dendrites. This mainly due to the zinc ions could preferentially nucleate and grow at these zincophilic sites rather than growing randomly across the electrode surface, just like there is an orderly guide point in the chaotic crowd, so that zinc ions “queue up” for deposition. Zincophilic functional groups can also form a close interface structure with the surface of zinc metal. These structures can enhance the compatibility between the zinc anode and the electrolyte, reducing the resistance of the interface. A favorable interfacial connection confers advantages: it mitigates polarization induced by poor contact and facilitates smoother migration of Zn^2^⁺ at the interface. This is analogous to a well‐paved road, where Zn^2^⁺—acting as “vehicles”—can migrate more rapidly and stably. Zincophobic functional groups are usually steric or chemically stable groups, for example, some fluorine‐containing functional groups or macromolecular polymer groups.^[^ ^117^, ^118^ ^]^ These functional groups can form a structure similar to a “barrier” on the surface of the zinc anode, which can physically or chemically prevent zinc ions from getting too close to the electrode surface and forming disorderly deposits. From the perspective of space, it is like holding up a “small umbrella” on the surface of the zinc electrode, and zinc ions are not easy to directly contact the electrode. Zincophobic functional groups can also forestall side reactions on the surface of the zinc anode caused by other impurities in the electrolyte or solvent molecules. It is difficult for impurities and solvent molecules to come into contact with the zinc anode owing to these functional groups occupying the active site on the electrode surface, thereby reducing the occurrence of side reactions, including zinc's reaction with water and other unstable components in the electrolyte. Such a mechanism just like putting on a layer of “protective clothing” for the zinc anode in the complex electrolyte environment. Zincophilic functional groups cooperate with each other to stabilize zinc deposition process, reduce side reactions and extend battery life.

Amikacin sulfate (AS), as one kind of aminoglycoside molecules, was also introduced into the aqueous zinc‐ion battery electrolyte as additives recently.^[^ ^112^ ^]^ Amikacin is rich in zincophilic groups, which can change the hydrogen bond network and solvation structure in the electrolyte through interaction with aqueous solution and Zn^2+^. In addition, amikacin can be adsorbed on the surface of zinc anode to reduce the occurrence of side reactions. As can be seen from Figure 9f, the contact angle of the electrolyte on the Zn surface decreases from 76.98° to 67.38°, indicating that amikacin can improve the wettability through adsorption. During the charging and discharging process, a protective layer formed by decomposing amikacin can further regulates deposition behavior of Zn^2+^ at the interface (Figure 9e). Other similar aminoglycoside additives have also been shown to significantly improve electrochemical performances.^[^ ^112^ ^]^

#### 3.2.3 Organic–Inorganic Hybrid Interphase Design

**Figure 10:**
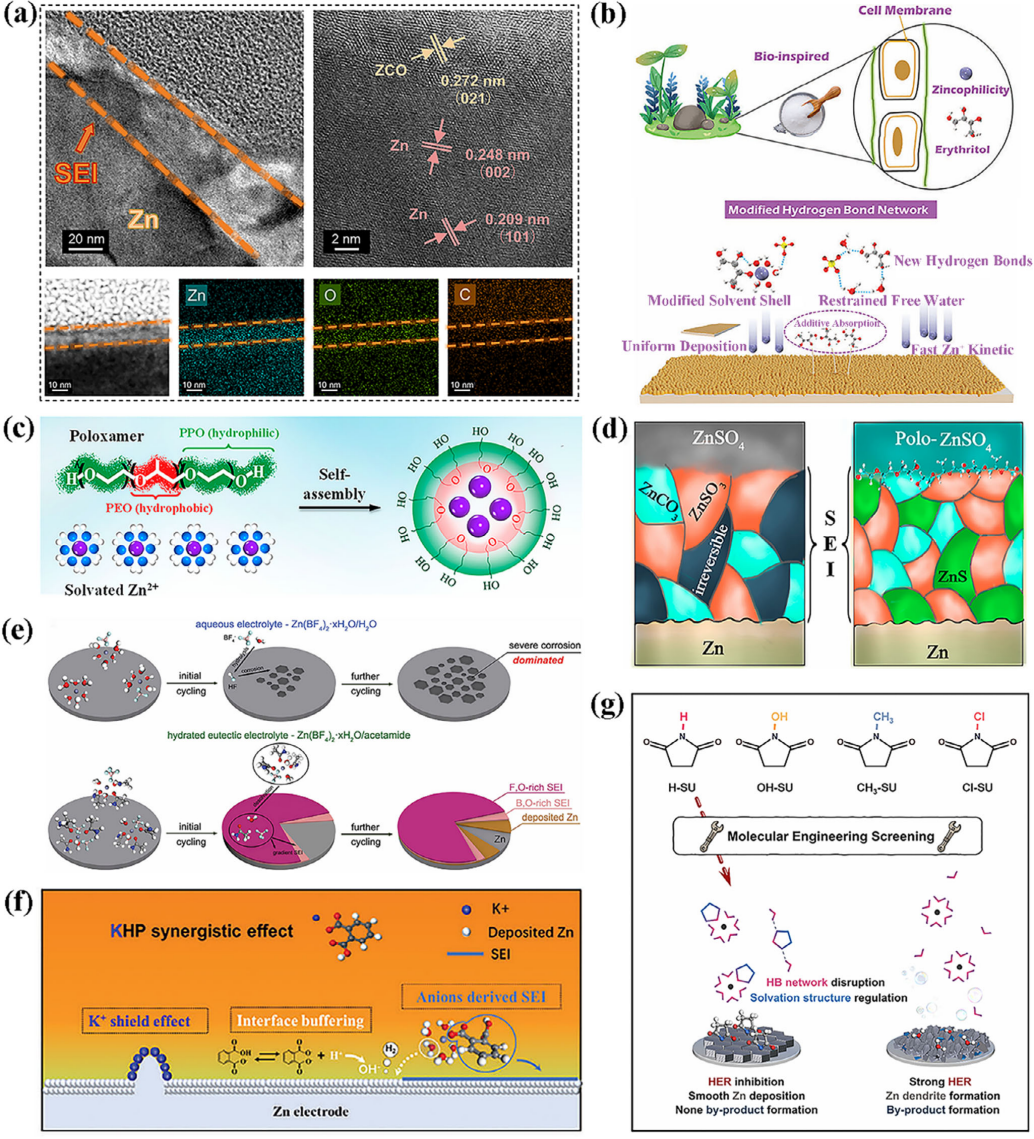
a) HR‐TEM image of the crystalline Zn and ZCO SEI, and the EDX mapping of Zn, O, and C.^[^ ^119^ ^]^ Copyright 2025, Springer Nature. b) The Ert additive and the related working mechanisms on Zn anode.^[^ ^120^ ^]^ Copyright 2024, John Wiley and Sons. c) Schematic illustration of the self‐assembled pre‐solvation process of poloxamers with Zn^2+^ ions. d) The SEI under the ZnSO_4_ and Polo‐ZnSO_4_ electrolytes.^[^ ^121^ ^]^ Copyright 2024, American Chemical Society. e) Schematic illustration of the Zn^2+^ solvation structure and corresponding interfacial features in aqueous and hydrated eutectic electrolyte.^[^ ^122^ ^]^ Copyright 2024, John Wiley and Sons. f) Schematic illustration of the synergistic effect of the cations and anions of KHP‐50 electrolyte on the Zn surface.^[^ ^123^ ^]^ Copyright 2024, John Wiley and Sons. g) Schematic illustration of the design and role of the H‐SU additive in ZMBs.^[^ ^124^ ^]^ Copyright 2024, The Royal Society of Chemistry.

Additionally, in the research on electrolytes for zinc metal batteries, there exists a core design concept centered on organic‐inorganic hybrid structures. By leveraging the high ionic conductivity of inorganic materials and the flexibility of organic materials, a synergistic effect is achieved, which significantly enhances the cycling performance of batteries. The organic compoundtrans‐fumaric acid (FU) can ionize to form divalent FU^2−^, which combines with inorganic Zn^2^⁺ to form a hybrid solvated structure [Zn^2^⁺(H_2_O)_4_FU^2−^]. Concurrently, an inorganic Zn_5_(CO_3_)_2_(OH)_6_ (ZCO) SEI layer forms on the Zn surface (**Figure** **10**a). The flexibility of the organic component effectively mitigates cracking issues during SEI cycling.^[^ ^119^ ^]^ The organic compound erythritol (Ert) accomplishes this by forming a complex with inorganic Zn^2^⁺ via its multiple hydroxyl groups, converting the solvation structure from [Zn(H_2_O)_6_]^2^⁺ to [Ert‐Zn(H_2_O)_5_]^2^⁺. This modification not only preserves the high conductivity of Zn^2^⁺ but also enhances interfacial stability through the dynamic adsorption layer of the organic component (Figure 10b).^[^ ^120^ ^]^ The organic poloxamer forms micelles via self‐assembly: its PEO core adsorbs inorganic Zn^2^⁺, while its PPO shell layer sequesters free water (Figure 10c), thereby constructing a pre‐solvated shell‐hybrid structure. Upon decomposition, it further generates an inorganic ZnS SEI layer (Figure 10d), realizing a dual function of ionic conductivity and interfacial protection.^[^ ^121^ ^]^ The organic compound acetamide forms a hydrated eutectic with inorganic Zn(BF_4_)_2_. Acetamide imparts flexibility to the system and inhibits the hydrolysis of BF_4_^−^. Concurrently, an inorganic gradient SEI—characterized by a B, O‐rich inner layer (facilitating high mass transfer) and a F, O‐rich outer layer (providing strong protection)—is generated in situ (Figure 10e).^[^ ^122^ ^]^ The organic phthalate anion (HP^−^) forms a cooperative hybrid system with the inorganic cation K⁺. The HP^−^ reconfigures the solvation structure of Zn^2^⁺, while K⁺ exerts an electrostatic shielding effect; collectively, these two components optimize the electrode‐electrolyte interface (Figure 10f).^[^ ^123^ ^]^ The organic compound succinimide (H‐SU) forms a “hybrid interface consisting of an organic adsorption layer and an inorganic inorganic inorganic ion‐conducting layer” by coordinating with inorganic Zn^2^⁺ via its amine groups and regulating the hydrogen bond network through its carbonyl groups (Figure 10 g).^[^ ^124^ ^]^ These designs achieve a significant enhancement in cycling performance through the synergistic effect of “the inorganic phase ensuring rapid ion transport and the organic phase improving interfacial flexibility and stability”.

### 3.3 Sodiophilic–Sodiophobic SEI

Sodium metal batteries (SMBs) have garnered significant attention owing to their high theoretical capacity (1166 mAh g^−1^), low reduction potential (−2.71 V vs SHE), and abundant sodium resources.^[^ ^133^ ^]^ Despite the high theoretical capacity and low electrochemical potential of sodium metal anodes, they suffer from challenges such as oxidation, unstable SEI layers, and dendrite growth. Specifically, the drastic volume changes and dendrite formation during sodium stripping and deposition processes lead to poor Coulombic efficiency and safety hazards. To tackle these issues, various strategies have been proposed, including the fabrication of artificial SEI films, the design of novel sodium current collectors, and electrolyte engineering. The application of these strategies has significantly enhanced the performance of the battery (**Table** **3**). Among these, electrolyte engineering is considered the simplest and most feasible approach, which aims to construct stable SEI films by regulating electrolyte components.

**Table 3:** Comparison of the Effects of In situ/Artificial SEI in Different Systems on the Electrochemical Performance of Sodium Metal Batteries.

Types	Additive	Symmetrical cycle	Refs.
**Additive**	Vinylpyrrolidone	2,000 h (5 mA cm^−2^, 1 mAh cm^−2^)	[125]
	No additive	No more than 500 h	
	N, S‐CDs	1,200 h (1 mA cm^−2^, 1 mAh cm^−2^)	[126]
	No additive	600 h	
	Sodium difluoro‐oxalate borate	800 h (1 mA cm^−2^, 1 mAh cm^−2^)	[127]
	No additive	350 h	
	Perfluorobenzene	300 h (1 mA cm^−2^, 1 mAh cm^−2^)	[52]
	No additive	150 h	
	N, N‐dimethyltrifluoromethane‐sulfonamide	6,000 h (3 mA cm^−2^, 6 mAh cm^−2^)	[128]
	No additive	400 h	
	pentafluoro(phenoxy)cyclotriphosphazene	1,400 h (0.1 mA cm^−2^, ‐40 °C)	[129]
	No additive	No more than 300 h	
**Artificial SEI**	Na/VN‐S	700 h (1 mA cm^−2^, 1 mAh cm^−2^)	[130]
	Bare Na	No more than 100 h	
	Ag_2_Na/Ag/Na_3_PO_4_	more than 1,600 h (0.5 mA cm^−2^, 1 mAh cm^−2^)	[131]
	Bare Na	No more than 100 h	
	OHTAPQ@Na	more than 1,500 h (2 mA cm^−2^, 2 mAh cm^−2^)	[132]
	Bare Na	No more than 400 h	

#### 3.3.1 The Core Mechanism of Sodiophilic–Sodiophobic SEI

**Figure 11:**
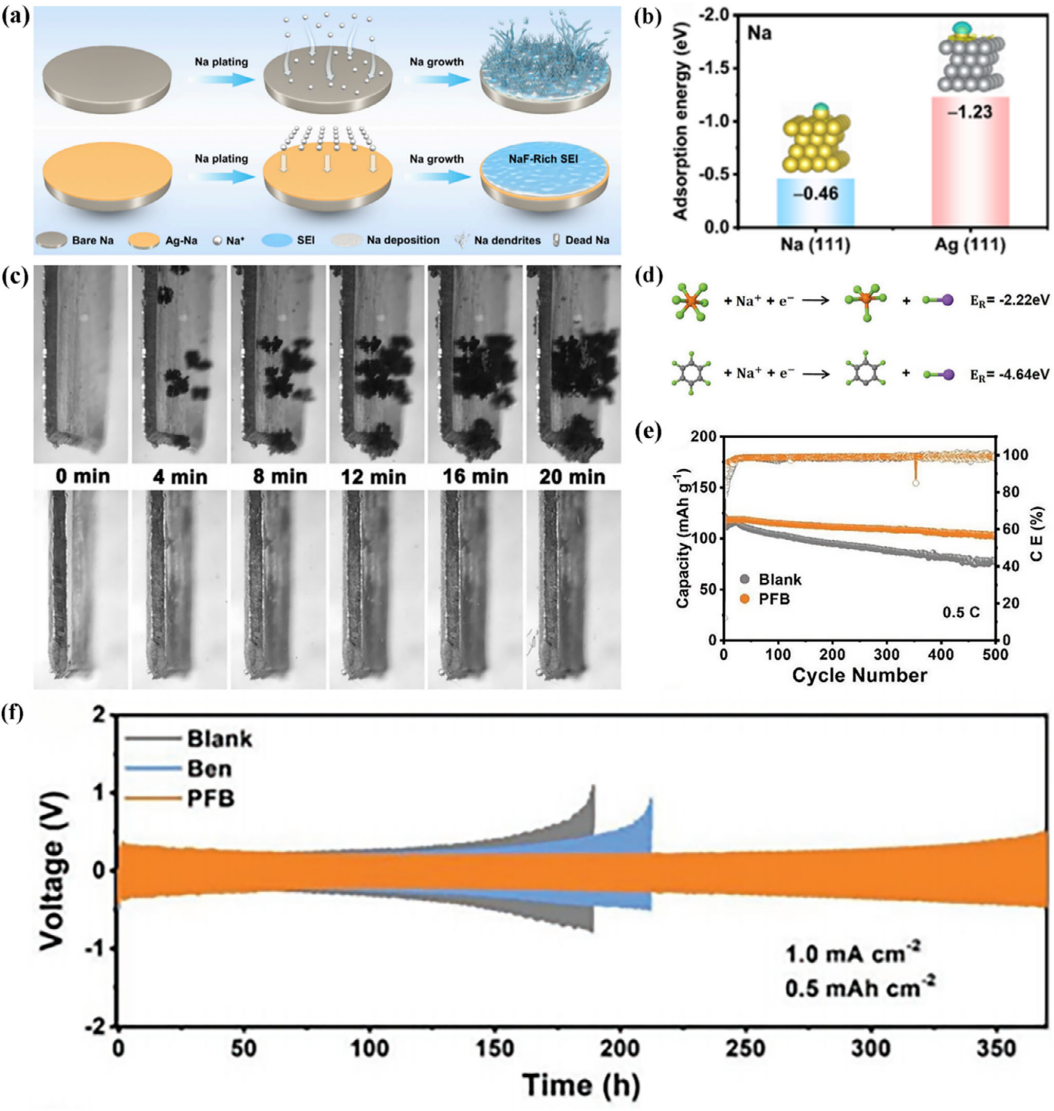
a) Schematic illustration of Na deposition behavior on bare Na and Ag‐Na. b) The calculated differential charge density and adsorption energy of Na at Ag and Na surfaces, in which yellow regions represent charge gain and cyan regions represent charge loss.^[^ ^20^ ^]^ Copyright 2025, American Chemical Society. c) In situ photos of Na plating in blank and 1 wt% PFB‐contained electrolytes at 1 mA cm^−2^. d) Reaction mechanism of reductive decomposition of PF_6_^−^ and PFB to generate NaF. e) Cycling capability of Na||Na_3_V_2_(PO_4_)_2_O_2_F batteries. f) Cycling performance of Na||Na symmetric cells with various electrolytes with 0.5 mAh cm^−2^.^[^ ^52^ ^]^ Copyright 2023, John Wiley and Sons.

Sodiophilic sites (e.g., metal nanoparticles, polar functional groups) act as preferential adsorption sites during the initial deposition stage by reducing the nucleation overpotential of sodium ions. The research team led by Prof. Kexing Song fabricated a silver (Ag)‐modified layer on the surface of sodium metal via an in situ chemical reduction method (**Figure** **11**a). Density functional theory (DFT) calculations reveal that Ag exhibits a higher Na adsorption energy than Na itself (Figure 11b), indicating strong affinity of the Ag layer for Na.^[^ ^20^ ^]^ This energetic advantage facilitates uniform nucleation of sodium ions on the interfacial layer, thereby preventing dendrite initiation induced by localized electric field concentration.

Sodiophobic regions typically consist of inorganic phases with high ionic conductivity and electronic insulation (e.g., NaF, B_2_O_3_). Their high interfacial energy and mechanical strength can effectively block dendrite penetration and inhibit electrolyte infiltration. The research team led by Prof. Chunlei Zhu from Hunan University induced the formation of a NaF‐enriched layer using a perfluorobenzene (PFB) additive. Calculations of the reaction energies for NaF formation via the reductive decomposition of PF_6_^−^ and PFB reveal that PFB decomposes into NaF more readily than PF_6_^−^, as indicated by its lower reaction energy (−4.64 eV < −2.22 eV) (Figure 11d). Thus, the NaF component is derived from the decomposition of both PFB and PF_6_^−^. To visualize the sodium deposition process, in situ optical microscopy was employed to record the growth of sodium dendrites. In the blank group, sodium dendrites grew uncontrollably and rapidly as deposition proceeded. In contrast, the sodium metal in the PFB group maintained a smooth surface throughout the process, with no dendrites observed, indicating uniform sodium deposition (Figure 11c).^[^ ^52^ ^]^

The sodiophilic sites and sodiophobic regions form a gradient structure featuring “inner‐layer induction and outer‐layer restriction”, thereby achieving synergistic optimization of ionic transport and mechanical stability.

#### 3.3.2 Electrolyte Additives

In SMBs, the formation process of the SEI shares similarities with that in lithium metal batteries, but notable differences also exist. Sodium metal exhibits high chemical reactivity, resulting in complex chemical reactions between the electrode and electrolyte during the initial charging cycle. Solvent molecules in the electrolyte—such as ether compounds—undergo reductive decomposition on the sodium metal surface. Concurrently, anions from sodium salts react with sodium metal to form corresponding compounds. These initial reaction products gradually deposit on the electrode surface, forming a primary SEI. As charging proceeds, reaction products accumulate and interact with one another, eventually forming a continuous SEI layer. Unlike lithium metal batteries, the larger atomic radius of sodium influences the structure and properties of the SEI. Inorganic components in the inner layer of the SEI in SMBs may exist as various sodium salts, with their compactness and stability differing from those of the inner layer in lithium metal battery SEIs. The types and contents of organic components in the outer layer also vary depending on electrolyte composition, thereby affecting ionic transport performance. Electrolyte additives play a crucial role in constructing sodiophilic‐sodiophobic SEIs: by incorporating specific additives into the electrolyte, the composition and structure of the SEI can be tailored to achieve sodiophilic‐sodiophobic functionalities.

Certain inorganic additives can regulate the sodium ion deposition process. For instance, fluorine‐containing inorganic additives in sodium metal batteries can be preferentially adsorbed onto the sodium metal surface, increasing the initial sites for sodium ion deposition. These sites help guide uniform sodium ion deposition, inhibit sodium dendrite growth, and simultaneously modulate the nucleation overpotential of sodium deposition. Fluorine‐containing additives such as PFB and sodium bis(fluorosulfonyl)imide (NaFSI) undergo preferential decomposition to form NaF nanocrystals, thereby increasing the proportion of inorganic components in the SEI.

The research team led by Chunlei Zhu proposed utilizing PFB as an electrolyte additive to improve the cycle life of sodium metal batteries.^[^ ^52^ ^]^ In this study, PFB was introduced into the electrolyte as an additive. Taking advantage of its conjugated fluorinated ring structure, PFB induces the reconstruction of the solvation sheath and facilitates the preferential decomposition of PF_6_^−^ into NaF, thus stabilizing the sodium anode surface. Furthermore, compared to the solvents EC and propylene carbonate (PC), PFB exhibits a higher highest occupied molecular orbital (HOMO) energy level and a lower lowest unoccupied molecular orbital (LUMO) energy level. This characteristic also enables it to preferentially decompose into NaF on both electrode surfaces. Benefiting from the increased NaF content in the SEI, the Na||Na symmetric cell achieved stable sodium plating/stripping for over 350 h at a current density of 1 mA cm^−2^ (Figure 11e). Furthermore, the Na||Na_3_V_2_(PO_4_)_2_O_2_F full cell retained 88.8% of its initial capacity after 500 cycles (Figure 11f).

**Figure 12:**
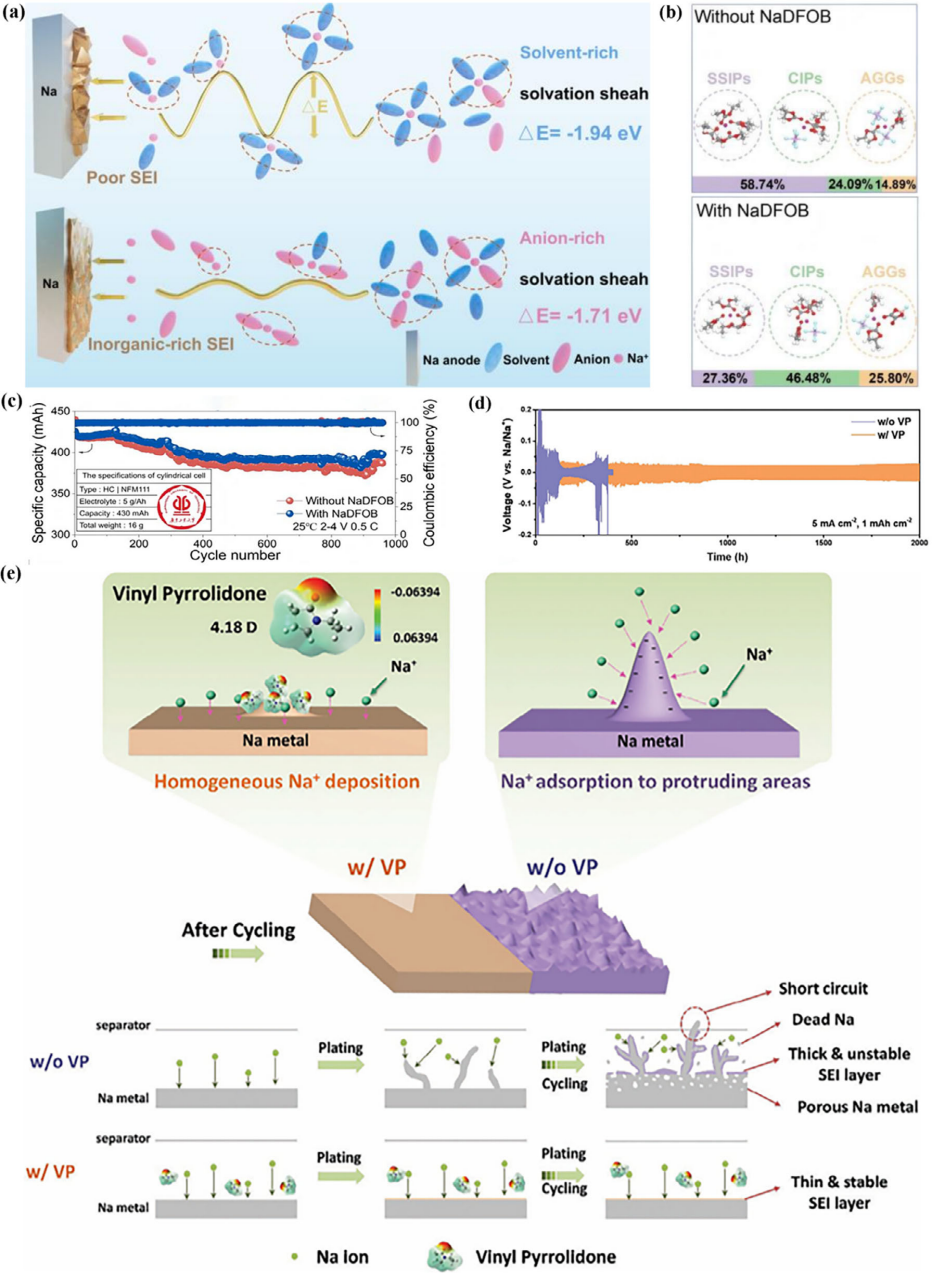
a) Schematic diagram of SEI formation rich in inorganic substances. b) Percentages of SSIPs, CIPs, and AGGs and representative primary solvation structures of Na^+^ in BE and BE‐NaDFOB electrolytes obtained by MD simulations. c) The long‐term cycling performance of the HC||NMF111 cylindrical cell at 0.5 C.^[^ ^127^ ^]^ Copyright 2025, Elsevier. d) Na‐Na symmetric cell tests using an electrolyte with and without VP: cycling performance of the Na metal anode at 5 mA cm^−2^ illustrated through charge‐discharge curves. e) Schematic depicting the Na ion plating process in Na metal electrodes with (w/) and without (w/o) the VP additive.^[^ ^125^ ^]^ Copyright 2024, John Wiley and Sons.

Sodium difluoro(oxalato)borate (NaDFOB), as an ester‐based electrolyte additive, regulates the solvation structure of the pristine electrolyte by enhancing coordination with Na⁺, resulting in an anion‐aggregated Na⁺ solvation sheath. The rapid desolvation of Na⁺ facilitates uniform deposition of sodium ions on the anode via the SEI, forming an SEI layer composed of NaF and B_2_O_3_—characterized by excellent electronic insulation and high mechanical strength (**Figure** **12**a). Recently, the research team from Sun Yat‐sen University employed molecular dynamics simulations to reveal that the introduction of NaDFOB allows more anions in the solvation sheath to enter the first solvation shell. This leads to the formation of a weakly solvated state, where anions coordinate with Na⁺ to form contact ion pairs (CIPs) and aggregates (AGGs) (Figure 12b), while concurrently lowering the desolvation energy barrier.^[^ ^127^ ^]^ The hard carbon||Na_0_._6_[Ni_0_._2_Fe_0_._2_Mn_0_._6_]O_2_ (HC||NFM) full cell with NaDFOB additive retained 94.12% of its capacity after 1000 cycles under the rated operating voltage and 0.5 C charge/discharge conditions, which outperformed the blank group (Figure 12c).^[^ ^127^ ^]^

Vinylpyrrolidone (VP), as a polarizable molecular dipole electrolyte additive, exhibits excellent performance in SEI layer formation and sodium deposition regulation. The molecular dipole of VP not only enables uniform sodium deposition by preferentially adsorbing onto sodium defect sites but also facilitates the formation of a dense and durable SEI layer through its own preferential decomposition (Figure 12e).^[^ ^125^ ^]^ Notably, the VP additive significantly reduces the nucleation overpotential during sodium deposition. Under different current density conditions, the nucleation overpotential required for sodium deposition is notably decreased after VP addition, indicating that VP can lower the energy barrier during the deposition process and promote the smooth deposition and uniform growth of sodium. The surface flattening effect induced by the molecular dipole of VP, coupled with the stable SEI, enables the battery to operate stably over long periods under a high current density of 5 mA cm^−2^ (Figure 12d), thus avoiding the voltage fluctuations and short‐circuit issues commonly observed in traditional batteries.

### 3.4 Potassiophilic–Potassiophobic SEI

Potassium metal batteries have emerged as promising candidates for large‐scale energy storage, owing to their high theoretical capacity (686 mAh g^−1^), extremely low reduction potential (−2.93 V vs SHE), and abundant natural resources.^[^ ^142^ ^]^ However, their practical application is severely hindered by the high reactivity of potassium metal anodes with electrolytes, interfacial issues arising from the large ionic radius of K⁺ (1.38 Å), and uncontrollable dendrite growth. The stability and ionic transport properties of the SEI are pivotal to addressing these challenges. The in situ/artificial SEI strategy is an important approach for optimizing battery performance (**Table** **4**). In recent years, designing “potassiophilic‐potassiophobic” synergistic strategies via electrolyte additives and constructing artificial SEI layers have become key research directions for optimizing SEI structures.

**Table 4:** Comparison of the Effects of In situ/Artificial SEI in Different Systems on the Electrochemical Performance of potassium Metal Batteries.

Types	Additive	Symmetrical cycle	Refs.
**Additive**	CDs	more than 1000 h (0.1 mA cm^−2^)	[134]
	No additive	No more than 115 h	
	Triphenyl phosphate	1000 h (0.5 mA cm^−2^, 0.5 mAh cm^−2^)	[135]
	No additive	No more than 600 h	
	Adiponitrile	400 h (4 mA cm^−2^, 4 mAh cm^−2^)	[60]
	No additive	200 h	
	amyl‐triphenyl‐phosphonium bromide	4200 h (0.1 mA cm^−2^, 0.1 mAh cm^−2^)	[136]
	No additive	No more than 700 h	
**Artificial SEI**	BS@K	2240 h (0.5 mA cm^−2^, 0.5 mAh cm^−2^)	[137]
	Bare K	No more than 250 h	
	Na_3_OCl/Na_3_Bi@K	4000 h (0.5 mA cm^−2^, 0.5 mAh cm^−2^)	[138]
	Bare K	No more than 300 h	
	SC‐Al_2_O_3_@K	800 h (1 mA cm^−2^, 1 mAh cm^−2^)	[139]
	Baseline‐K	No more than 300 h	
	IOHL‐K	more than 2100 h (0.5 mA cm^−2^, 0.5 mAh cm^−2^)	[140]
	Bare K	No more than 250 h	
	K‐Plated in 50% FEC	more than 1400 h (0.4 mA cm^−2^, 0.2 mAh cm^−2^)	[141]
	Bare K	No more than 550 h	
	SC‐1600@K	more than 2000 h (0.5 mA cm^−2^, 0.5 mAh cm^−2^)	[21]

#### 3.4.1 The Core Mechanism of Potassiophilic–Potassiophobic SEI

Potassiophilic sites (e.g., cyano groups (C≡N) in organic additives, metal nanoparticles, and alloy phases) serve as preferential adsorption sites during the initial potassium deposition stage, with their core function being the reduction of K⁺ nucleation overpotential.^[^ ^142^ ^]^ This strong potassium affinity enables potassiophilic regions to accumulate K⁺ at the interface, facilitating uniform nucleation of metallic potassium and suppressing dendrite initiation induced by local electric field concentration. Additionally, alloy phases commonly employed in artificial SEI design (e.g., Sn‐K and Bi‐K alloys) exhibit excellent potassium affinity. They can provide abundant nucleation sites to homogenize K⁺ flux and guide the ordered growth of potassium.

Potassiophobic regions are typically composed of inorganic phases with high ionic conductivity and electronic insulation, such as KF, K_2_CO_3_, and K_3_PO_4_. These components fulfill two critical functions: First, their high interfacial energy and dense structure can physically block potassium dendrite penetration, thereby preventing electrode short circuits. Second, their electronic insulation properties can inhibit the continuous reduction and decomposition of electrolyte molecules on the potassium surface, avoiding the formation of a thick, loose SEI layer that impedes ion transport.

Potassiophilic sites and potassiophobic regions collectively form a gradient structure characterized by “inner‐layer induction and outer‐layer restriction”. The inner potassiophilic layer regulates the initial deposition behavior of K⁺ to achieve uniform nucleation, while the outer potassiophobic layer provides mechanical support and ion transport channels. This synergistic effect comprehensively optimizes the ion transport efficiency and mechanical stability of the SEI. It effectively addresses critical issues of potassium metal anodes, including severe interfacial side reactions, significant volume changes, and uncontrollable dendrite growth, thereby laying a foundation for enhancing the cycling stability and safety of potassium metal batteries.

#### 3.4.2 Electrolyte Additives

Electrolyte additives act as a crucial means to regulate the properties of the SEI layer in potassium metal batteries. Different types of additives exhibit distinct action mechanisms, thus exerting significant impacts on battery performance.

**Figure 13:**
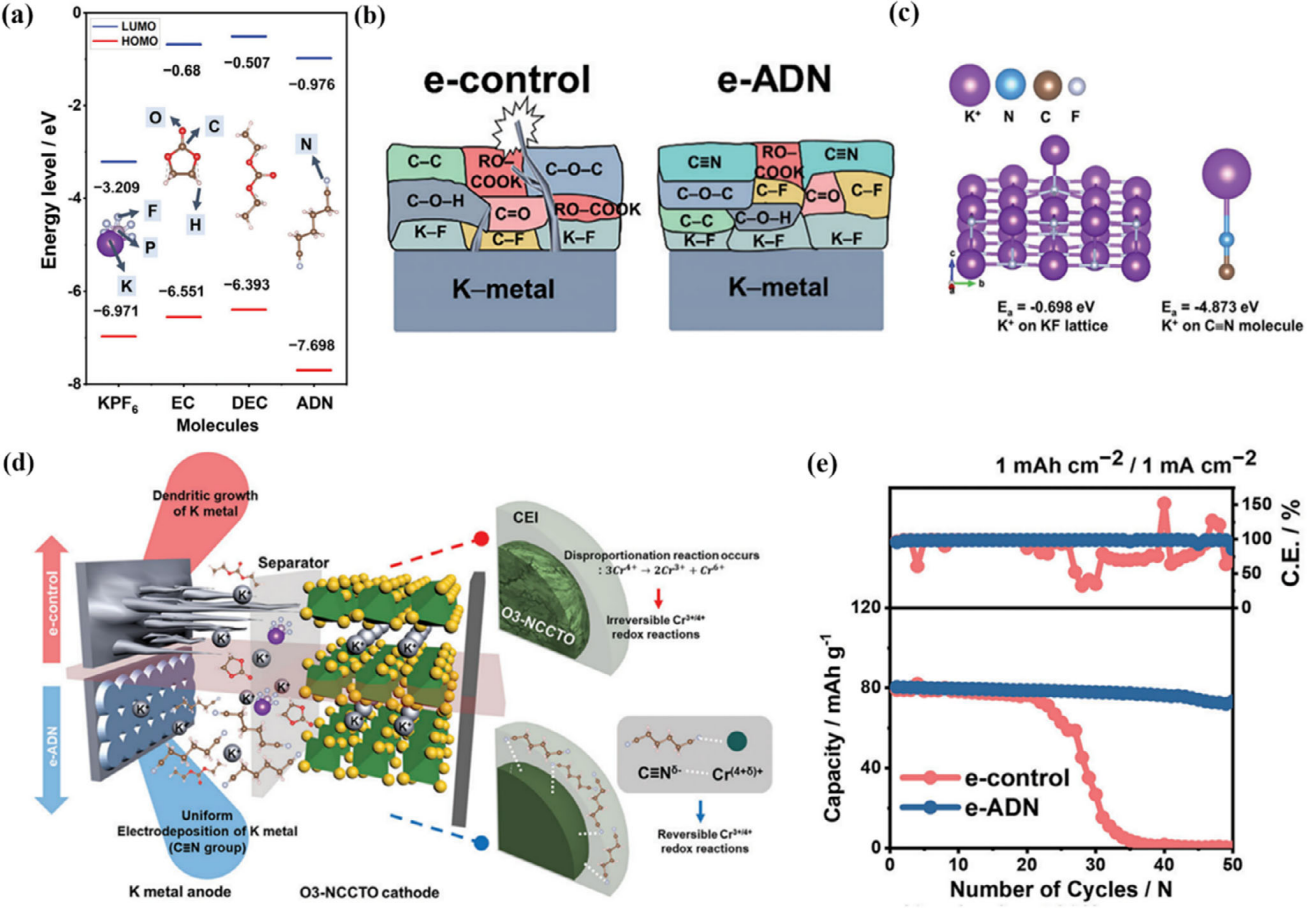
a) HOMO‐LUMO energy levels of the salt (KPF_6_), solvents (EC and DEC), and additive (AND). b) Schematic of morphology of SEI layers formed on potassium metal anode in contact with (left) e‐control and (right) e‐AND. c) DFT calculations for the adsorption energies of (left) K^+^ on KF and (right) on the C≡N group. d) Schematic of the dual‐functional effect of ADN for K anode and O3‐NCCTO cathode. e) The cycling performance of the full battery in the electrolyte at a capacity of 1 mAh cm^−2^ and a current density of 1 mA cm^−2^.^[^ ^60^ ^]^ Copyright 2023, John Wiley and Sons.

ADN is a bifunctional electrolyte additive containing electron‐rich nitrile groups (C≡N). Owing to its low HOMO energy level (−7.698 eV), ADN is more resistant to oxidation compared to common carbonate solvents such as EC (−6.551 eV) and diethyl carbonate (DEC, −6.393 eV), thereby endowing the electrolyte with superior stability at high voltages. Furthermore, the LUMO energy level of ADN is −0.976 eV, which is lower than those of the carbonate solvents EC (−0.68 eV) and DEC (−0.507 eV) (**Figure** **13**a).^[^ ^60^ ^]^ This implies that ADN is more readily reduced on the surface of the potassium metal anode and can preferentially form a stable SEI layer prior to solvent molecules during the reduction process. The SEI layer formed via this preferential reduction contains potassiophilic C≡N groups, which are capable of forming potassiophilic compounds with potassium ions. These groups guide the uniform deposition of potassium ions, thereby inhibiting the growth of potassium dendrites (Figure 13b).^[^ ^60^ ^]^ DFT calculations reveal that the adsorption energy of potassium ions on the C≡N group (−3.8321 eV) is an order of magnitude higher than that on KF (−0.399 eV) (Figure 13c). This indicates that the C≡N group can adsorb potassium ions more effectively, thereby promoting the uniform deposition of potassium metal. Furthermore, the C≡N moiety in nitrile groups can form strong coordination bonds with oxidized transition metals, mitigating undesirable disproportionation reactions and enhancing the thermal stability of layered transition metal oxide cathodes. By modifying the chemical properties of the electrolyte, this additive improves the stability of both the anode‐electrolyte and cathode‐electrolyte interfaces, thereby boosting the electrochemical performance of potassium metal batteries. The bifunctional effect of ADN is more pronounced in full cells (Figure 13d). At a current density of 1 mA cm^−2^, the full cell using the control electrolyte exhibits unstable cycling behavior and eventually fails after the 30th cycle. In contrast, the full cell with the ADN‐containing electrolyte maintains a high capacity retention rate (98.6%) and stable CE over 50 cycles (Figure 13e).

#### 3.4.3 Construction of Artificial SEI Layers

An artificial SEI layer is deposited on the surface of potassium metal anodes to inhibit side reactions between potassium metal and the electrolyte, thereby enhancing the battery's stability and cycle life. The design principles for artificial interfacial layers focus on two key aspects: first, strong potassium affinity, which provides abundant nucleation sites and homogenizes the potassium‐ion flux to promote uniform potassium deposition; second, low catalytic activity, which reduces electrolyte decomposition and prevents the formation of a thick, loose SEI layer. The former would impede ion migration, while the latter would facilitate dendrite growth.

**Figure 14:**
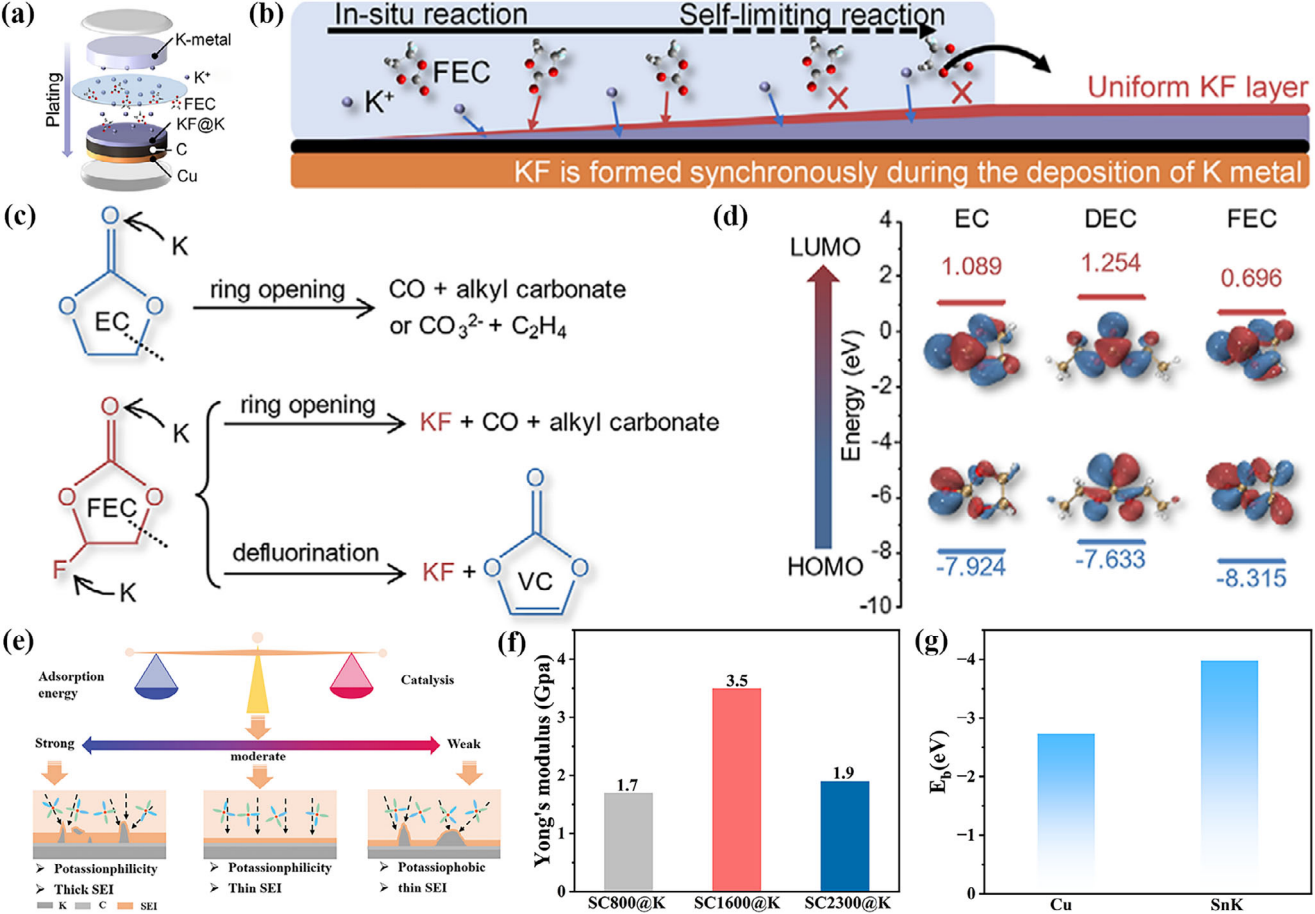
a) Schematic diagram for construction of KF‐rich SEI layer using electrodeposition. b) Schematic diagram of the interfacial reaction for the construction of a KF‐rich SEI layer on a K‐metal electrode by electrodeposition. c) Schematic equations for the reaction of K metal with EC and FEC respectively. d) The HOMO‐ LUMO energy levels of different compositions: EC, DEC, and FEC.^[^ ^141^ ^]^ Copyright 2025, John Wiley and Sons. e) The influence of adsorption energy and catalytic ability on K deposition and SEI formation. f) The corresponding value of Young's modulus after 5 cycles at 0.5 mA cm^−2^.^[^ ^21^ ^]^ Copyright 2024, John Wiley and Sons. g) Corresponding Eb for Cu and Sn‐K alloy.^[^ ^143^ ^]^ Copyright 2023, American Chemical Society.

Constructing a KF‐rich artificial SEI layer via electrodeposition is also an effective approach. During the electrodeposition process, an in situ reaction occurs between potassium metal and electrolyte additives (e.g., FEC), forming a uniform KF‐rich SEI layer (**Figure** **14**a,b).^[^ ^141^ ^]^ Kinetically, in terms of solvation number and initial desolvation behavior, FEC exhibits the highest coordination number, rendering it the most effective at releasing K⁺. Furthermore, compared with EC, the primary reaction products of FEC with potassium metal are potassium fluoride and vinylene carbonate (VC), rather than organic compounds. The generated VC further inhibits the reaction between potassium metal and EC (Figure 14c).^[^ ^141^ ^]^ Reduced decomposition of EC inhibits the precipitation of potassium hexafluorophosphate (KPF_6_), effectively alleviating electrolyte decomposition issues. Moreover, calculations of the LUMO and HOMO energy levels for each electrolyte component (Figure 14d) further verify that FEC is more readily reducible than EC and DEC, thereby facilitating the formation of a KF‐rich SEI. This SEI layer can effectively protect the electrode and suppress dendrite growth, thereby enhancing the stability and cycle life of potassium metal anodes.^[^ ^141^ ^]^

Furthermore, Professor Yuyan Yu's research group has successfully designed and fabricated a locally ordered carbon material (SC‐1600) as an artificial interfacial layer for potassium metal battery anodes.^[^ ^21^ ^]^ This material balances potassiophilicity and catalytic activity by optimizing defect content, thereby enabling uniform potassium ion deposition and the formation of a stable SEI layer (Figure 14e). Moreover, its high Young's modulus (Figure 14f) endows it with excellent mechanical stability, which effectively suppresses side reactions between the electrolyte and electrode materials while reducing dendrite formation.^[^ ^21^ ^]^ Furthermore, a pre‐passivation strategy entails coating copper foam with a Sn‐K alloy and an artificial SEI layer.^[^ ^143^ ^]^ The Sn‐K alloy, rich in oxygen and fluorine elements, exhibits high potassiophilicity and physicochemical stability. Its strong potassium binding energy (E_b_) facilitates the uniform nucleation and deposition of potassium atoms (Figure 14g), thereby inhibiting dendrite growth. Meanwhile, the spontaneously formed SEI layer suppresses parasitic reactions prior to the initial plating process. In subsequent cycles, the alloy exhibits a low Fermi energy (E_f_) of potassium fluoride, functioning as a durable and suitable barrier for redistributing potassium atoms.

### 3.5 Magnesiophilic–Magnesiophobic SEI

Rechargeable magnesium batteries, like other metal‐based batteries, have emerged as promising candidates for large‐scale energy storage, owing to their high theoretical volumetric capacity (3833 mAh cm^−3^), low reduction potential (−2.37 V vs SHE), and abundant magnesium resources.^[^ ^144^ ^]^ However, their practical application is severely hindered by interfacial issues arising from the high reactivity of magnesium metal anodes with electrolytes, the large ionic radius of Mg^2^⁺ (0.72 Å), and uncontrollable dendrite growth.^[^ ^145^, ^146^ ^]^ The stability and ion transport properties of the SEI are crucial to addressing these challenges. In recent years, developing “magnesiophilic‐magnesiophobic” synergistic strategies via electrolyte additive design and constructing artificial SEI layers have become key research directions for optimizing the SEI structure (**Table** **5**).

**Table 5:** Comparison of the Effects of In situ/Artificial SEI in Different Systems on the Electrochemical Performance of magnesium Metal Batteries.

Types	Additive	Symmetrical cycle	Refs.
**Additive**	SnF_2_	900 h (1 mA cm^−2^, 2 mAh cm^−2^)	[147]
	No additive	No more than 20 h	
	TFEB	400 h (0.5 mA cm^−2^, 0.5 mAh cm^−2^)	[22]
	No additive	No more than 320 h	
	COF‐1	800 h (2 mA cm^−2^)	[148]
	No additive	No more than 500 h	
	TPB	240 h (0.1 mA cm^−2^, 0.05 mAh cm^−2^)	[149]
	No additive	No more than 50 h	
	BrFB	7000 h (0.1 mA cm^−2^, 0.05 mAh cm^−2^)	[150]
	No additive	No more than 200 h	
	OctylBr	3600 h (0.5 mA cm^−2^, 0.5 mAh cm^−2^)	[151]
	No additive	/	
**Artificial SEI**	In/MgCl_2_	1500 h (3 mA cm^−2^, 1 mAh cm^−2^)	[152]
	Bare Mg	No more than 1000 h	
	B‐PC@Mg	3500 h (0.3 mA cm^−2^, 0.15 mAh cm^−2^)	[153]
	Bare Mg	No more than 1, 700 h	
	FRAB@Mg	2200 h (0.5 mA cm^−2^, 0.25 mAh cm^−2^)	[154]
	Bare Mg	No more than 100 h	

#### 3.5.1 Core Mechanism of Action of Magnesiophilic–Magnesiophobic SEI

**Figure 15:**
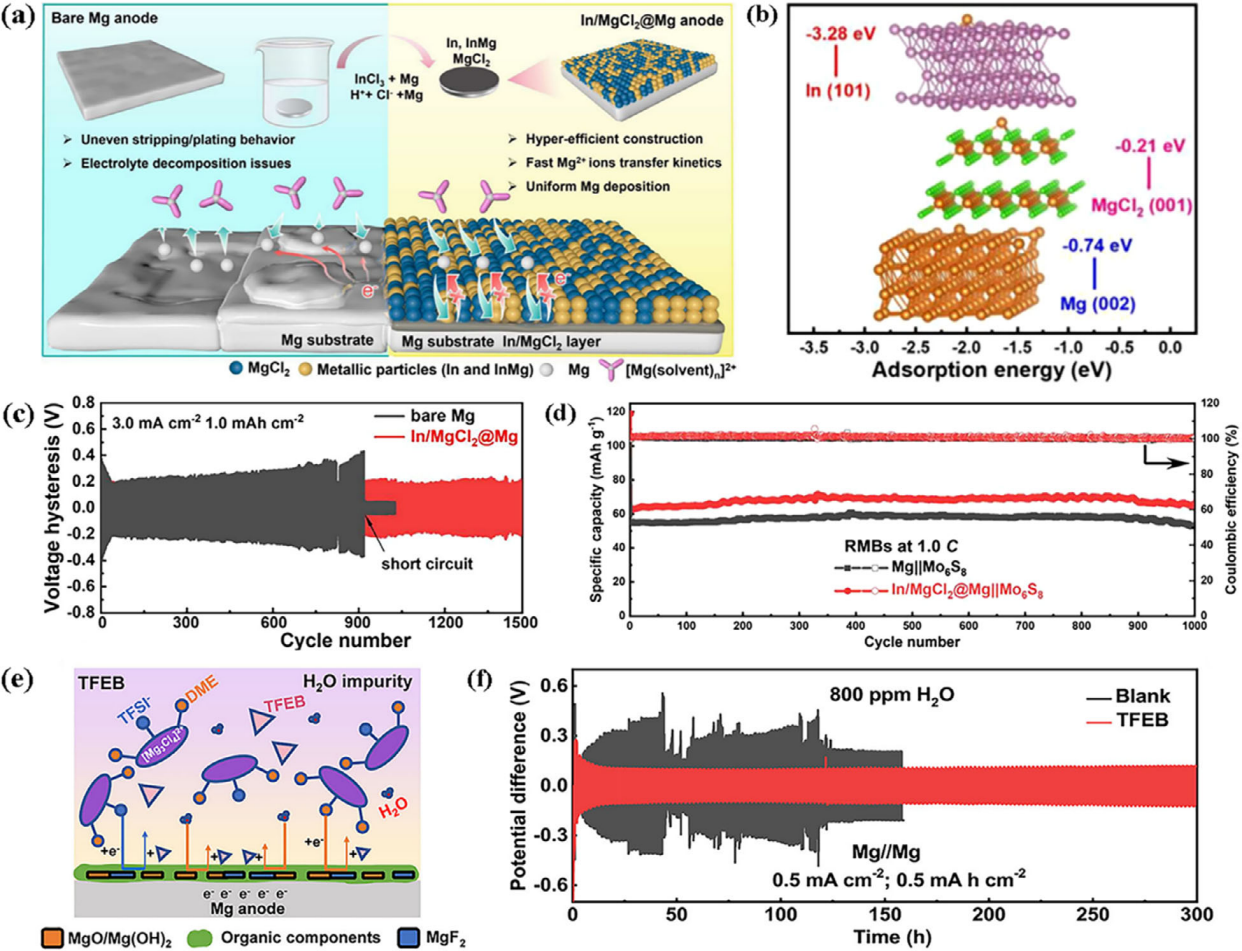
a) Schematic diagram of the Mg stripping/plating process on the surface of bare Mg anode (left) and In/MgCl_2_@Mg anode (right). b) Adsorption configurations and the corresponding adsorption energies of a Mg atom on the surfaces of In(101), MgCl_2_(001), and Mg(002). c) Cycling performance of symmetric cells with bare Mg and In/MgCl_2_@Mg anode at 3 mA cm^−2^ with 1 mA h cm^−2^. d) Long‐term cycling performance of the Mg||Mo_6_S_8_ full cells at a current density of 1 C.^[^ ^152^ ^]^ Copyright 2024, American Chemical Society. e) The SEI formation mechanism on the Mg anode in TFEB‐added electrolytes with 800 ppm H_2_O. f) Cycling performance of Mg||Mg cells at 0.5 mA cm^−2^ and 0.5 mA h cm^−2^ using blank and TFEB‐added electrolytes with the addition of 800 ppm H_2_O.^[^ ^22^ ^]^ Copyright 2024, John Wiley and Sons.

Magnesiophilic sites (e.g., metal nanoparticles, alloy phases) act as preferential adsorption sites during the initial deposition stage by reducing the nucleation overpotential of Mg^2^⁺. For example, an indium (In)‐modified layer can be constructed on the Mg metal surface via in situ chemical reduction, forming In and InMg alloy phases (**Figure** **15**a).^[^ ^152^ ^]^ DFT calculations reveal that the adsorption energy of Mg on In (−3.28 eV) is significantly lower than that on Mg itself (−0.74 eV) (Figure 15b), indicating strong affinity of the In layer for Mg. This energetic advantage facilitates uniform nucleation of Mg^2^⁺ on the interfacial layer, preventing dendrite initiation caused by localized electric field concentration. In the In/MgCl_2_ artificial SEI, the inner InMg alloy layer provides magnesiophilic sites to promote uniform nucleation, while the outer MgCl_2_ layer forms a dense inorganic barrier to suppress dendrite growth. This configuration enables the symmetric cell to achieve over 1500 cycles at 3 mA cm^−2^ (Figure 15c). Additionally, when paired with an Mo_6_S_8_ cathode, the In/MgCl_2_@Mg||Mo_6_S_8_ battery exhibits exceptional stability over 1000 cycles at 1C with no capacity decay (Figure 15d).

Magnesiophobic regions typically consist of inorganic phases with high ionic conductivity and electronic insulation (e.g., MgF_2_, MgCl_2_, B_2_O_3_). Their electronic insulation suppresses continuous electrolyte decomposition, while high ionic conductivity ensures efficient Mg^2^⁺ transport. For instance, the high interfacial energy and dense structure of MgF_2_ effectively block dendrite penetration, while the layered structure of MgCl_2_ provides channels for ionic migration.^[^ ^152^, ^155^ ^]^ Together, magnesiophilic sites and magnesiophobic regions form a gradient structure with “inner‐layer induction‐outer‐layer confinement,” achieving synergistic optimization of ionic transport and mechanical stability.

#### 3.5.2 Electrolyte Additive

**Figure 16:**
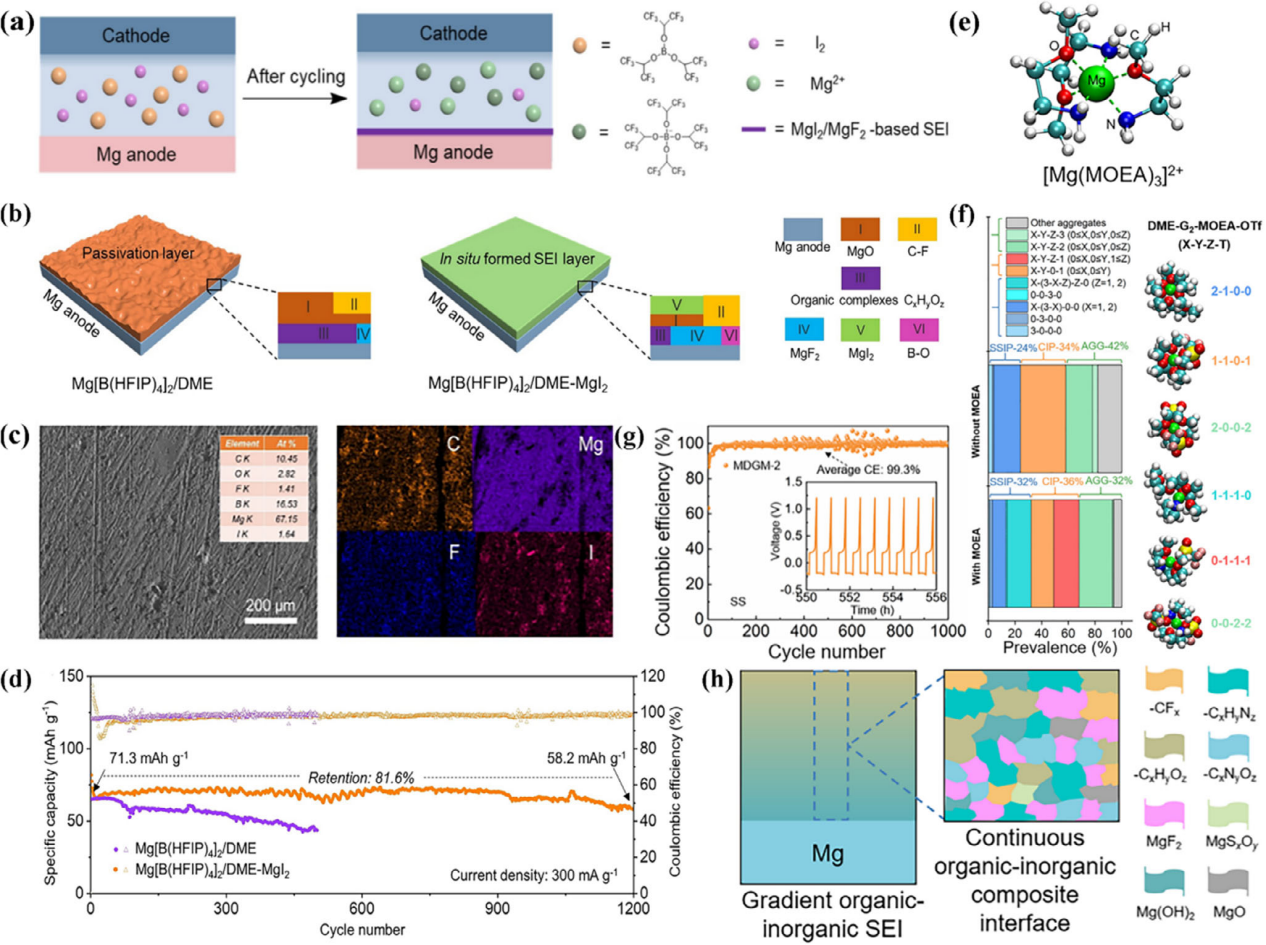
a) In situ preparation procedure of Mg[B(HFIP)_4_]_2_/DME‐MgI_2_ electrolyte and its bifunctional properties in generating the Mg[B(HFIP)_4_]_2_ and the MgI_2_/MgF_2_‐based SEI layer. b) Schematic representation of the surface passivation layer formed by pristine Mg[B(HFIP)_4_]_2_/DME and Mg[B(HFIP)_4_]_2_/DME‐MgI_2_ electrolyte on Mg anode. c) SEM and corresponding EDS mapping images of Mg anodes in Mg[B(HFIP)_4_]_2_/DME‐MgI_2_ electrolytes after 100 cycles. d) Corresponding cycling stability of Mg||Mo_6_S_8_ batteries utilizing Mg[B(HFIP)_4_]_2_/DME‐MgI_2_ and pristine Mg[B(HFIP)_4_]_2_/DME electrolytes at a current density of 300 mA g^−1^.^[^ ^155^ ^]^ Copyright 2024, John Wiley and Sons. e) The optimized solvation structures of [Mg(MOEA)_3_]^2+^ from B3LYP/6‐311G(d, p) DFT calculations. f) Solvation structure distribution analysis of the two systems. g) Cycling stability of the Mg||SS cell with the MDGM‐2 electrolyte at 1.0 mA cm^−2^ with 40 min in a single cycle. h) Schematic of the formed gradient organic‐inorganic SEI.^[^ ^156^ ^]^ Copyright 2023, Elsevier.

Boron‐based additives, such as TFEB,^[^ ^22^ ^]^ act as electron‐deficient additives. Their Lewis acidity enables reactions with electrolyte decomposition products (e.g., Mg(OH)_2_), reducing electron‐enriched MgO and MgF_2_ phases in the SEI while promoting the uniform distribution of magnesiophilic organic species (Figure 15e). With TFEB, the Mg||Mg symmetric cell maintains stable performance even with 800 ppm H_2_O, achieving a cycle life of 300 h—nearly twice that of the blank control group (Figure 15f).^[^ ^22^ ^]^ Additionally, the synergistic effect of iodine and tris(hexafluoroisopropyl) borate (B(HFIP)_3_) in situ generates a MgI_2_/MgF_2_ hybrid SEI (**Figure** **16**a,b).^[^ ^155^ ^]^ SEM observations show that the Mg anode surface remains relatively flat and smooth after 100 cycles in the Mg[B(HFIP)_4_]_2_/DME‐MgI_2_ electrolyte (Figure 16c). Furthermore, the Mg||Mo_6_S_8_ full cell based on this system exhibits excellent long‐cycle stability, retaining 81.6% of its capacity after 1200 cycles at 300 mA g^−1^ (Figure 16d).

2‐Methoxyethylamine (MOEA) coordinates with Mg^2^⁺ via its strongly nucleophilic amino group (‐NH_2_), thereby reconstructing the solvation shell (Figure 16e).^[^ ^156^ ^]^ Molecular dynamics simulations reveal that MOEA can enter the first solvation shell of Mg^2^⁺ to form CIPs (Figure 16f), promoting the formation of organic nitrogen‐rich species (magnesiophilic phase) in the inner SEI layer while inducing the generation of MgF_2_ and B_2_O_3_ (magnesiophobic phase) in the outer layer (Figure 16h). This strategy enables the Mg||Cu battery to cycle 1000 times at 1 mA cm^−2^ with a Coulombic efficiency maintained at 99.3% (Figure 16 g).^[^ ^156^ ^]^

#### 3.5.3 Construction of Artificial SEI

**Figure 17:**
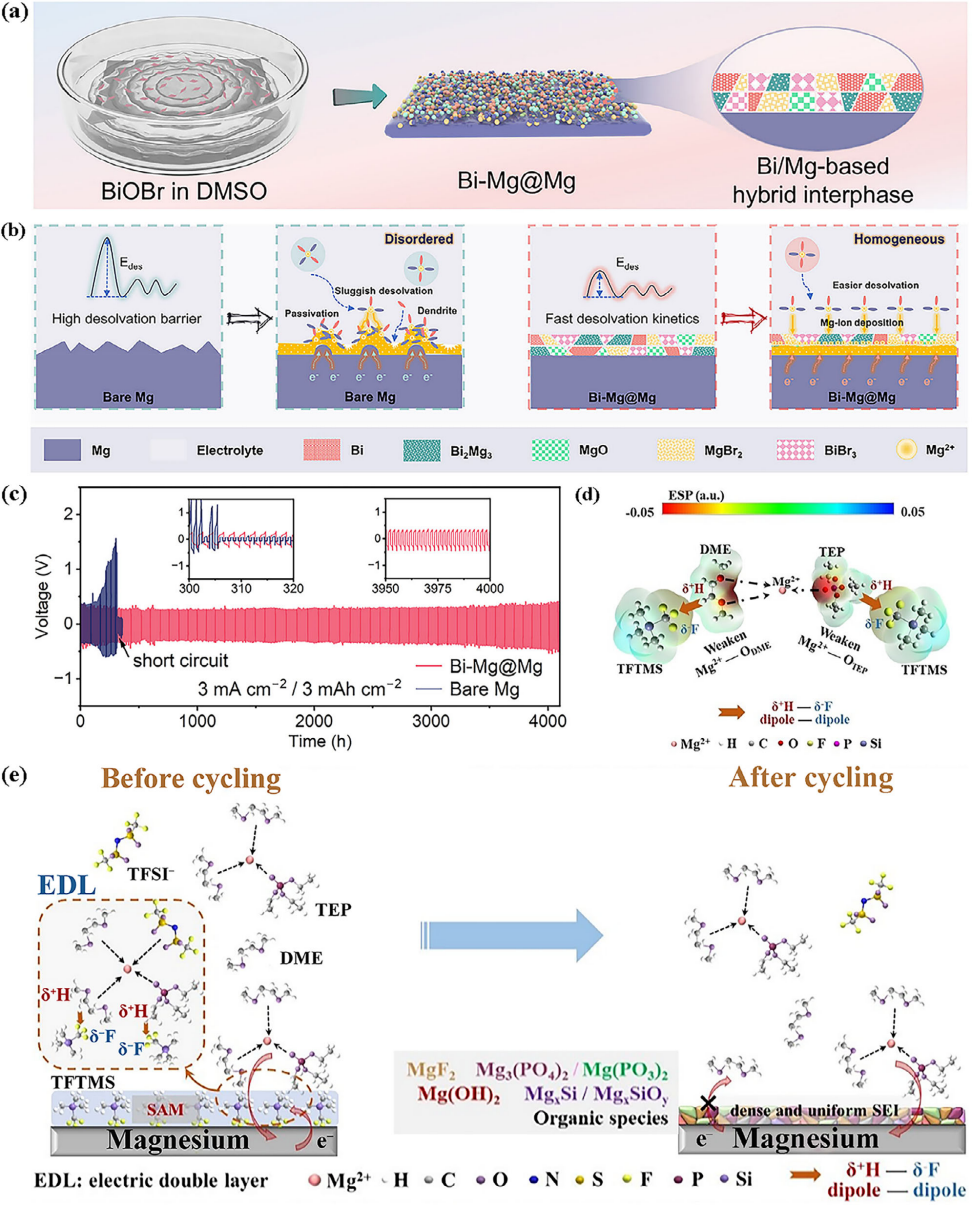
a) Schematic illustration of the preparation of Bi‐Mg@Mg through a quasi‐solid‐solid redox reaction. b) Schematic illustration of the Mg plating/stripping behaviors on bare Mg and Bi‐Mg@Mg electrodes. c) long cycling performance ofthe bare Mg and Bi‐Mg@Mg electrodes.^[^ ^82^ ^]^ Copyright 2025, John Wiley and Sons. d) The effect of TFTMS on dipole‐dipole interactions among surrounding solvent molecules (DME, TEP) and their coordination with Mg^2+^. e) The mechanism diagram of TFTMS additives to construct SAM and modulate SEI nanostructure.^[^ ^157^ ^]^ Copyright 2024, John Wiley and Sons.

A hybrid interface is constructed via a displacement reaction between metal salts and Mg foil. For instance, immersing Mg foil in a BiOBr suspension forms a Bi/Mg‐based hybrid SEI through a solid‐solid redox reaction (**Figure** **17**a): the inner layer, consisting of Bi and Bi_2_Mg_3_ alloy (magnesiophilic phase), facilitates rapid Mg^2+^ nucleation, while the outer layer—composed of MgO, MgBr_2_, and BiBr_3_ (magnesiophobic phase)—inhibits electrolyte corrosion (Figure 17b).^[^ ^82^ ^]^ This SEI enables the Mg||Mg symmetric cell to cycle for over 4100 h under 3 mA cm^−2^ and 3 mAh cm^−2^ (Figure 17c) and exhibits excellent compatibility with Mo_6_S_8_ and S cathodes.^[^ ^82^ ^]^

Additionally, Fan Zhengqing's group proposed a self‐assembled monolayer (SAM) strategy,^[^ ^157^ ^]^ where trifluoromethyltrimethylsilane (TFTMS) self‐assembles into an ordered molecular layer on the Mg surface via electron‐withdrawing groups (‐CF_3_). This layer weakens solvent‐Mg^2+^ coordination through dipole‐dipole interactions (Figure 17d), enhances interfacial adsorption of anions (e.g., TFSI^−^), and induces formation of a uniform magnesiophilic‐magnesiophobic gradient SEI (Figure 17e). The SAM layer weakens solvent‐Mg^2+^ coordination and promotes oriented MgF_2_ growth, leading to a 4‐fold improvement in SEI electronic insulation (R_E_/R_I_ = 13.7),^[^ ^157^ ^]^ which significantly suppresses side reactions.

## 4 Function of SEI in Extreme Conditions

However, the practical performance of batteries is significantly compromised under extreme conditions, such as those encountered in deep space exploration, ocean expeditions, and polar missions. These harsh environments not only pose new challenges for batteries but also induce unique failure mechanisms.^[^ ^158^ ^]^ To address these issues, the design of SEI films and their impact mechanisms on battery performance under extreme conditions are summarized as follows.

### 4.1 High Voltage Battery

In high‐voltage environments, side reactions between battery electrode materials and electrolytes tend to intensify. A well‐designed SEI can effectively suppress decomposition—particularly during high‐voltage charging—reduce gas generation inside the battery, and thereby enhance battery safety.^[^ ^159^ ^]^ A suitable SEI also helps maintain the structural stability of electrode materials: it prevents structural collapse or pulverization caused by side reactions with the electrolyte during repeated charge‐discharge cycles.^[^ ^160^, ^161^ ^]^ The structure and composition of the SEI can endow batteries with unique properties. Under high voltage, inorganic components in the SEI block electron conduction, thereby inhibiting oxidative electrolyte decomposition.^[^ ^6^ ^]^ These inorganic components exhibit high electrochemical stability and retain their chemical state under high voltage. Meanwhile, organic components in the SEI provide a certain degree of flexibility, buffering volume changes of the electrode material during charge‐discharge processes. This flexibility reduces SEI rupture and regeneration caused by volume fluctuations, thereby lowering internal resistance and helping maintain battery performance under high voltage.

**Figure 18:**
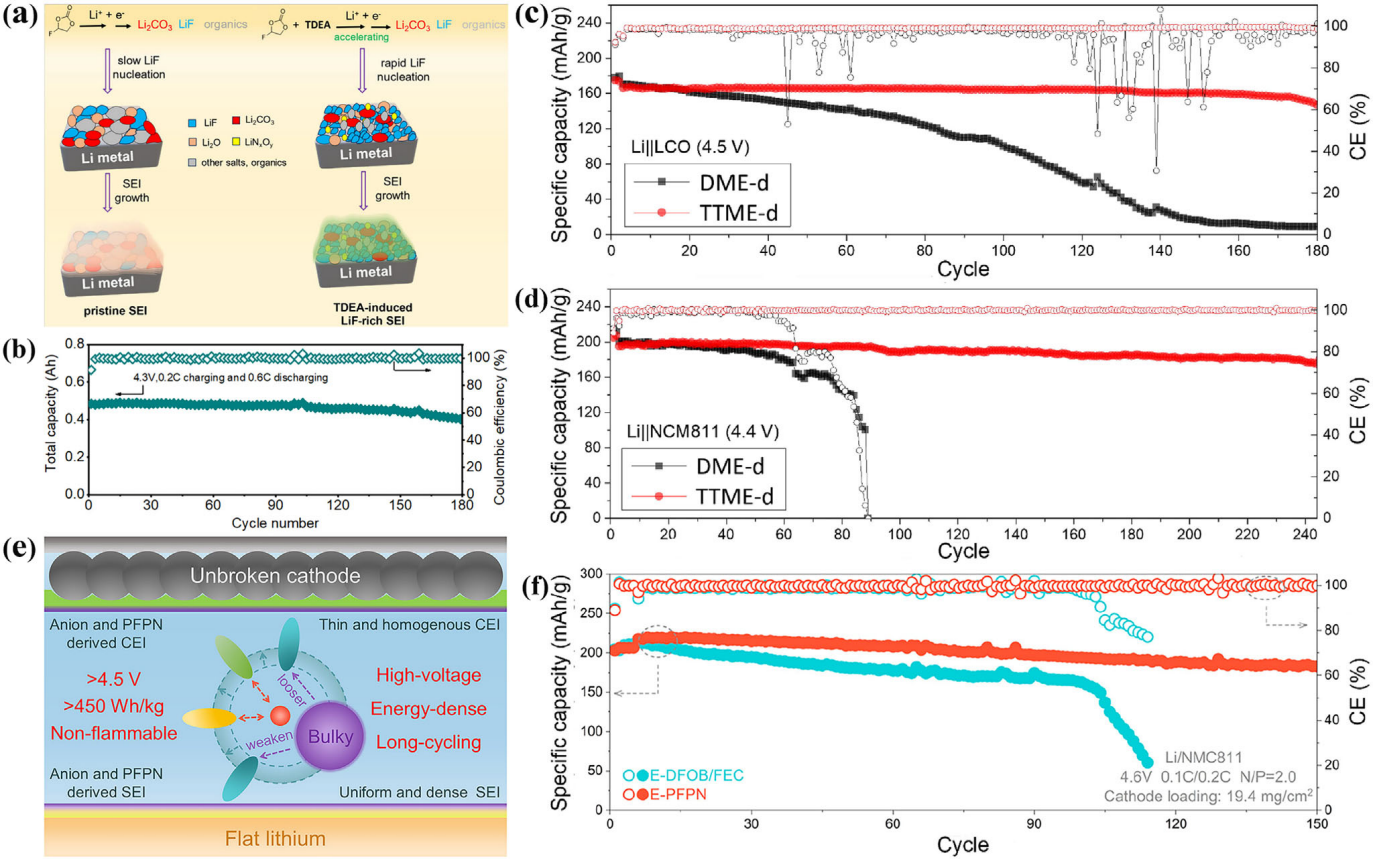
a) Schematic diagram of the LiF formation and SEI structural evolution in FEC‐based carbonate electrolytes. b) The performance of 347 Wh kg^−1^ Li|NCM811 pouch cells enabled by TDEA‐BE.^[^ ^160^ ^]^ Copyright 2024, American Chemical Society. c) Cycling performance of Li|LCO (4.5 V) cells. d) Cycling performance of Li|NCM811 (4.4 V) cells.^[^ ^162^ ^]^ Copyright 2024, Elsevier. e) Schematic diagram of the electrochemical process of the Li|NMC811 battery in E‐PFPN. f) Cells were cycled between 3.0 and 4.6 V at a 0.1 charge rate and a 0.2 C discharge rate.^[^ ^23^ ^]^ Copyright 2023, American Chemical Society.

To address this issue, Xia et al. proposed the use of a trace double‐salt electrolyte additive (TDEA), which accelerates the decomposition of FEC to generate LiF, improves LiF distribution, and induces its premature precipitation—ultimately forming a LiF‐rich SEI on lithium anodes (**Figure** **18**a).^[^ ^160^ ^]^ A 500 mAh pouch cell exhibited good cyclability (180 cycles) under practical harsh conditions with a cutoff voltage of 4.3 V (Figure 18b). The highly fluorinated ether molecule 1,1,1‐trifluoro‐2‐[(2,2,2‐trifluoroethoxy)methoxy]ethane (TTME), used as a co‐solvent electrolyte, enables the formation of a double‐layer SEI on lithium metal anodes.^[^ ^162^ ^]^ One layer is rich in crystalline components to enhance SEI mechanical strength, while the other contains higher concentrations of organic components to improve flexibility. A 1.4 M LiFSI‐TTME:DME (4:1) electrolyte (TTME‐d)—where DME is 1,2‐dimethoxyethane—was compared with a 1.4 M LiFSI‐DME electrolyte (DME‐d). The Li||NCM811 battery retained 85% capacity after 240 cycles at a cutoff charge voltage of 4.4 V, and the Li||LCO battery maintained 90% capacity after 170 cycles at 4.5 V (Figure 18c,d).^[^ ^162^ ^]^ Another strategy involves solvation tuning via molecular steric effects to create “bulk coordination” structures. Bulky ethoxy(pentafluoro)cyclotriphosphazene (PFPN) efficiently coordinates with Li⁺; its steric hindrance weakens the coordination ability of conventional solvents (e.g., DME), resulting in a loose Li⁺ solvation structure with increased Li⁺‐anion and Li⁺‐PFPN coordination.^[^ ^23^ ^]^ The designed electrolyte yielded an inorganic‐rich SEI (Figure 18e), and the Li||NCM811 full battery retained 84.1% capacity over 150 cycles at an ultrahigh voltage of 4.6 V (Figure 18f). Additionally, a series of trifluoromethanesulfonamide solvents were designed to simultaneously regulate steric hindrance and electronic properties by systematically controlling the N‐terminal ring size.^[^ ^163^ ^]^ This enhances contact ion pair formation for anion‐derived SEI. The LiFSI 1‐azacyclobutane trifluoromethanesulfonamide electrolyte achieves an optimal balance of steric and electronic effects, with high oxidation stability up to 5 V.

**Figure 19:**
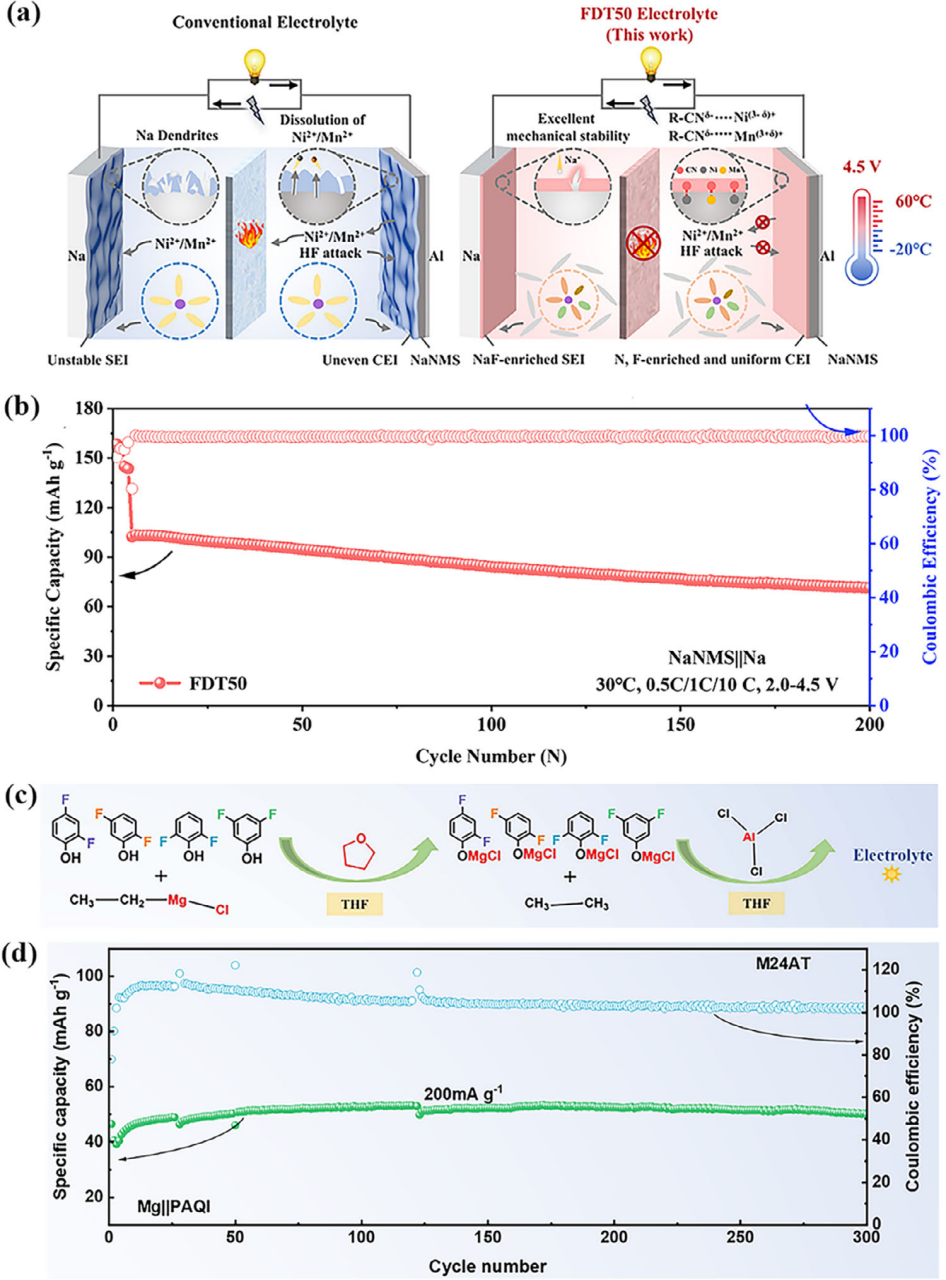
a) Schematic representation of solvation structures and electrochemical properties of conventional and FDT50 electrolytes. b) Cycling performance and CE of NaNMS||FDT50||Na at 10 C.^[^ ^164^ ^]^ Copyright 2025, American Chemical Society. c) Synthesis steps for the electrolytes of M24AT, M25AT, M26AT, and M35AT. (d) Cycling performance. Mg||PAQI full cell performance in M24AT electrolyte.^[^ ^165^ ^]^ Copyright 2024, Elsevier.

In sodium metal batteries, an electrolyte system featuring both high‐voltage compatibility and broad temperature‐range adaptability is proposed through the coupling of cyanide, fluorine, and ether functional groups. This electrolyte forms an ultrathin, homogeneous N/F‐rich interface (incorporating CN^−^, CNO^−^, NaF, etc.) at the positive electrode, which inhibits the dissolution of TMn⁺ and the oxidative decomposition of the electrolyte. Meanwhile, a NaF‐rich SEI is formed on the negative electrode, enhancing the uniformity of sodium deposition and the ability to suppress dendrite growth (**Figure** **19**a). The optimized electrolyte enables the sodium metal battery to deliver an initial capacity of 103.4 mAh g^−1^ at a high voltage window of 2–4.5 V under a 10 C charge/discharge rate, along with an average Coulombic efficiency of 99.63% after 200 cycles (Figure 19b). It also enables the battery to operate stably across a wide temperature range of −20 to 60 °C. Furthermore, the high fluoride content and cyano groups synergistically reduce the electrolyte's flammability, thereby mitigating the risk of thermal runaway.^[^ ^164^ ^]^ In the field of magnesium metal batteries, a 2,4‐difluorophenylmagnesium electrolyte (designated as M24AT) featuring superior oxidation stability has been designed (Figure 19c). M24AT demonstrates enhanced capability in stabilizing the fluorinated SEI. Electrochemical characterization reveals that this electrolyte exhibits superior oxidation stability (3.9 V vs Mg/Mg^2^⁺), outperforming all previously reported magnesium‐phosphine electrolytes. Furthermore, it shows excellent compatibility with a diverse range of cathode materials. When employed in the Mg||PAQI full cell, stable cycling performance is achieved under a maximum full‐electrochemical operating voltage of 3.0 V (Figure 19d). This thus marks the first successful application of this class of electrolytes under such a high‐voltage operating condition.^[^ ^165^ ^]^

### 4.2 Fast‐Charging Battery

SEI design is critical for fast‐charging batteries. During rapid charging, Li⁺ undergoes rapid deposition and dissolution at the electrode, placing stringent demands on SEI stability.^[^ ^166^, ^167^, ^168^ ^]^ An ideal SEI for fast charging should minimize Li⁺ migration resistance at the interface,^[^ ^169^, ^170^ ^]^ enabling rapid transport through the SEI layer to the electrode interior. For instance, a battery with a thin, uniform SEI can complete charging in less time than one with a thick, non‐uniform SEI. Furthermore, an optimal SEI design should enhance battery safety during fast charging. High‐current charge‐discharge processes generate significant heat; poor thermal stability of the SEI may cause rupture, leading to safety hazards. Thus, a well‐designed SEI can effectively dissipate heat and maintain structural stability at high temperatures, preventing thermal runaway induced by fast charging.

Furthermore, the pore structure and chemical composition of the SEI layer can effectively regulate Li⁺ migration rates. The SEI contains channels that facilitate Li⁺ transport, with the size, distribution, and connectivity of these channels depending on the SEI composition. Specifically, channels formed by lithium salt decomposition products exhibit a low Li⁺ migration energy barrier, enabling rapid ion transport. Simultaneously, the electronic insulation of the SEI prevents unnecessary electron transfer between the electrode and electrolyte, mitigating side reactions. This is particularly critical during fast charging, as the heightened electrochemical activity within the battery amplifies the risk of such reactions.

**Figure 20:**
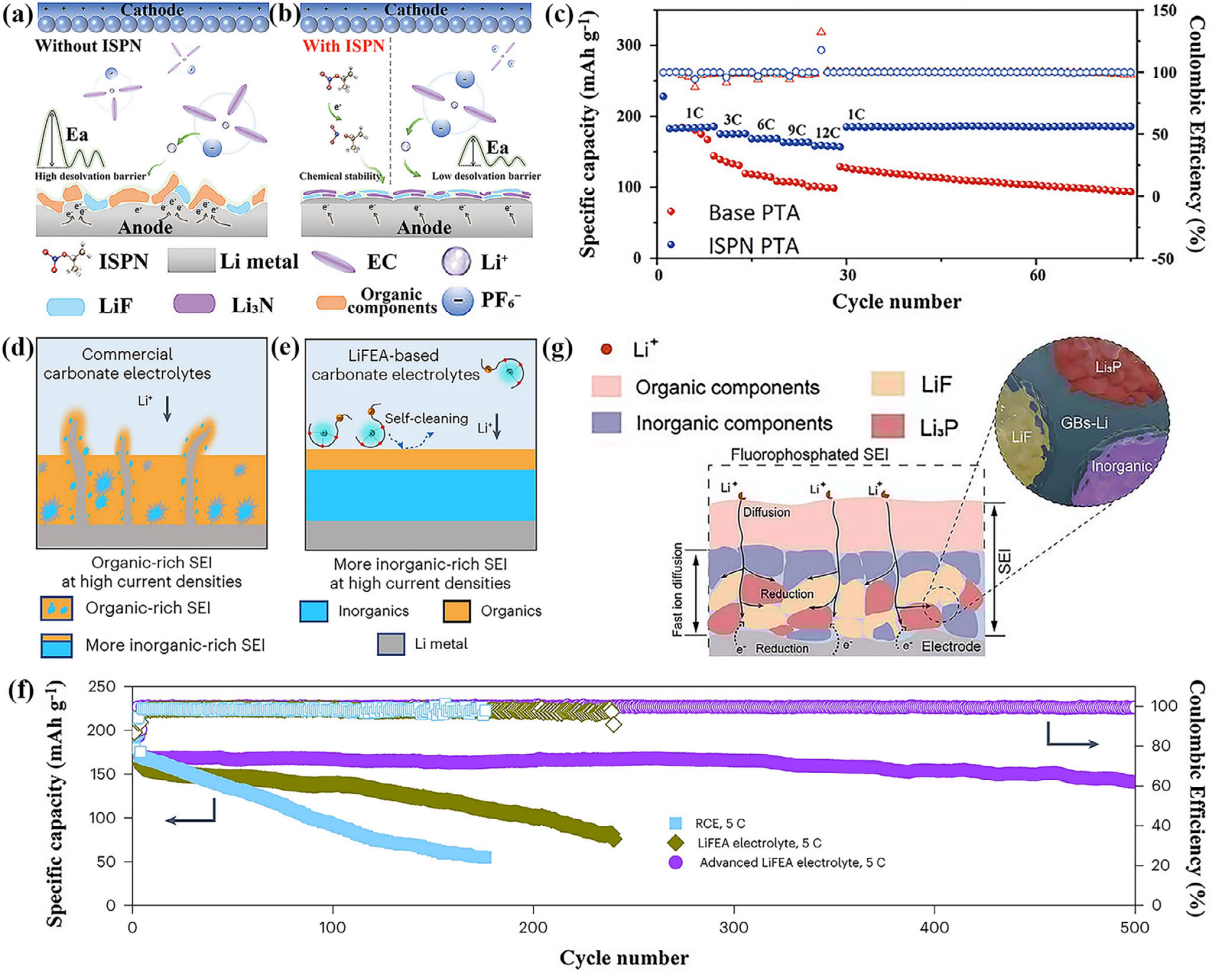
a,b) Schematic diagram of the interaction mechanism of ISPN. c) Rate performance in NCM622|Base‐PTA|Li and NCM622|ISPN‐PTA|Li cells.^[^ ^168^ ^]^ Copyright 2024, John Wiley and Sons. d,e) LiFEA‐based electrolytes enabling a more inorganic‐rich SEI layer and dendrite‐free Li deposition by self‐cleaning action, compared with commercial carbonate electrolytes. f) Cycle performance with RCE, LiFEA electrolyte, and advanced LiFEA electrolyte under 5C.^[^ ^171^ ^]^ Copyright 2023, Springer Nature. g) Li can quickly cross the low diffusion barrier grain boundary in the form of BGs‐Li and then deposit on the electrode in an orderly manner.^[^ ^167^ ^]^ Copyright 2024, John Wiley and Sons.

A new class of nitrogen‐containing additive, isopropyl nitrate (ISPN), has recently been shown to be miscible with ester solvents, enabling the formation of a chemically stable LiF‐Li_3_N composite SEI with high ionic conductivity.^[^ ^168^ ^]^ The interaction mechanism of ISPN is illustrated in **Figure** **20**a,b. Notably, the LiFePO_4_ (LFP)||ISPN‐PTA||Li cell exhibits excellent cycling stability and fast‐charging capability, maintaining stable operation for 850 cycles at a 10 C rate (Figure 20c).

Traditional carbon‐based electrolytes are highly corrosive to lithium metal. To address this, an asymmetric lithium salt—lithium 1,1,1‐trifluoro‐N‐[2‐[2‐(2‐methoxyethoxy)ethoxy)]ethyl]methanesulfonamide (LiFEA)—has been designed, featuring a folded pseudocrown ether‐like molecular geometry.^[^ ^171^ ^]^ This salt endows carbonate electrolytes with a high apparent donor number and lithium‐ion transference number, while driving a self‐cleaning mechanism in the SEI (Figure 20d,e)—thus enhancing compatibility with lithium metal anodes even at high current densities. Three electrolytes were tested for battery cycling: a commercial electrolyte (1.0 M LiPF_6_ in EC/DEC, 1:1 v/v) as the reference carbonate electrolyte (RCE); a LiFEA electrolyte (0.1 M LiFEA added to RCE) to evaluate the effect of LiFEA alone; and an advanced LiFEA electrolyte (0.1 M LiNO_3_ + 0.1 M LiFEA added to RCE). At 5 C, the advanced LiFEA electrolyte delivered a high specific capacity of 141 mAh g^−1^, with 83.5% capacity retention after 500 cycles (Figure 20f). Both the bulk electrolyte and SEI derived from the advanced LiFEA electrolyte facilitated faster Li⁺ transport compared to RCE.

Another strategy involves introducing a fluorophosphate SEI with abundant, fast ion‐diffusion inorganic grain boundaries (LiF/Li_3_P).^[^ ^167^ ^]^ This fluorophosphonate SEI was constructed using a sol electrolyte containing porous lithium nanoparticles modified with highly dispersed phosphorus‐containing functional groups, revealing the presence of electrochemically active lithium in these non‐nucleating fast ion‐diffusion grain boundaries (BGS‐Li) (Figure 20g). This design ensures the stability of Li||NCM811 batteries at a fast‐charging rate of 5 C for over 1000 cycles. As discussed earlier, the electrochemical performance of fluorophosphonate SEI on lithium metal was further validated.

**Figure 21:**
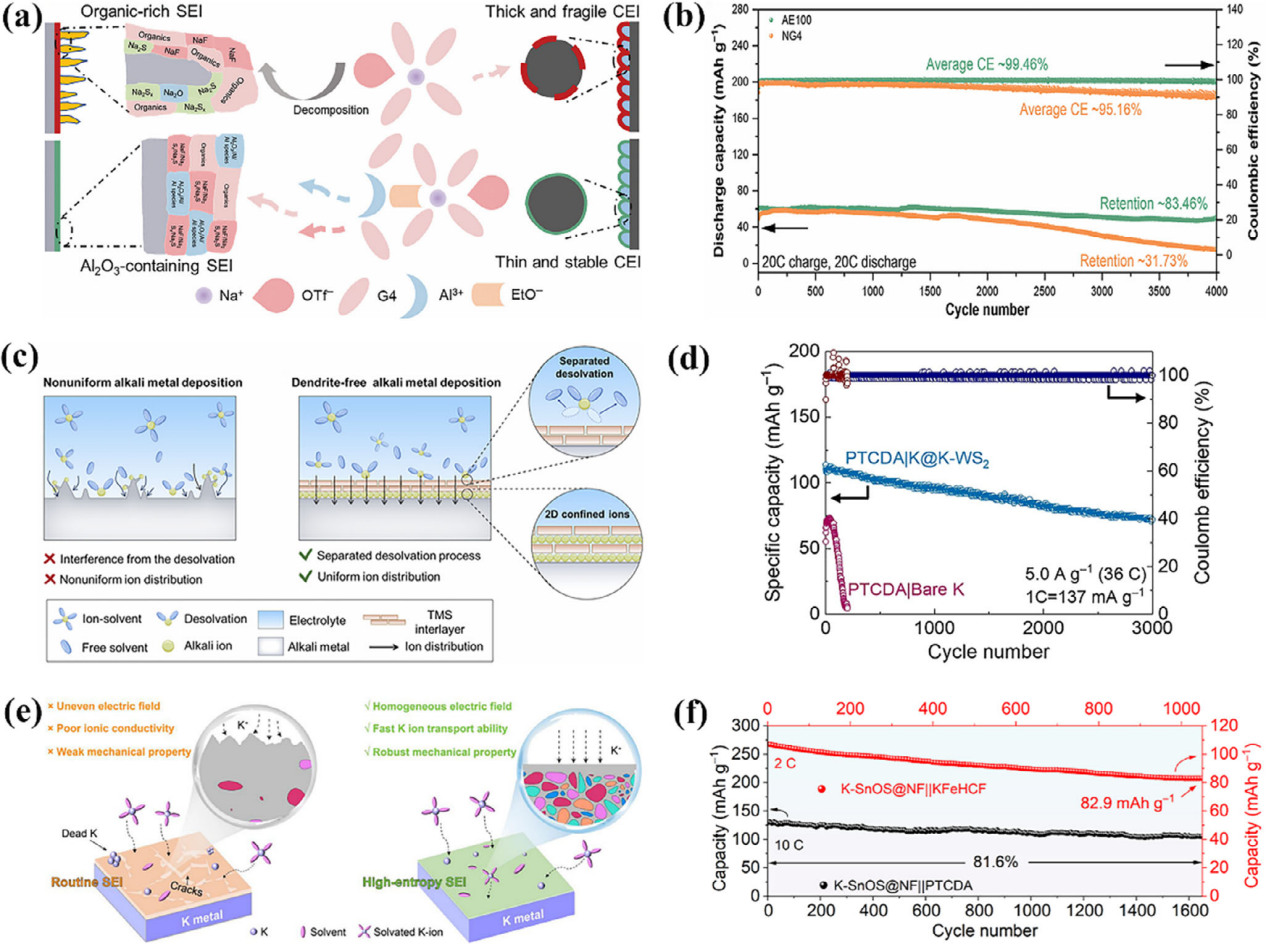
a) Schematic diagram of the formation mechanism of the SEI and CEI with Al(EtO)_3_ added in the electrolyte. b) Long‐term cycle stability of a Na|NVP full cell at 20 C (1 C = 117.6 mAh g^−1^) with the two electrolytes.^[^ ^172^ ^]^ Copyright 2024, John Wiley and Sons. c) Schematic images of nonuniform alkali metal deposition inferenced by the desolvation process and dendrite‐free alkali metal deposition by 2D confinement of desolvated ions. d) Cycling performance of PTCDA|bare K and PTCDA|K@K‐WS_2_ full cells at 5.0 A^−1^ (36 C).^[^ ^173^ ^]^ Copyright 2025, American Chemical Society. e) Schematic illustration of different types of SEIs on the metallic K anode. f) Cycling performance of the two types of PMB devices.^[^ ^56^ ^]^ Copyright 2025, John Wiley and Sons.

In sodium metal batteries, a straightforward strategy involving the introduction of aluminum alkoxide salts into conventional ether‐based electrolytes has been employed to simultaneously construct SEI and cathode electrolyte interphase (CEI) layers that are uniform, robust, and highly conductive to sodium ions (**Figure** **21**a). The Na||NVP full cell thus achieved exceptional performance, retaining 83.46% of its initial capacity after 4000 stable cycles at a high rate of 20 C (Figure 21b).^[^ ^172^ ^]^ In potassium metal batteries, 2D transition metal dichalcogenides are employed as interlayers, featuring high adsorption energy toward potassium ions and 2D confined channels for alkali metal ion transport (Figure 21c). When alkali metal ions traverse the 2D transition metal dichalcogenide interlayer, the desolvation process is effectively decoupled from metal deposition. This spatial separation further inhibits solvent‐metal side reactions during the deposition process, thereby facilitating the formation of a thin and uniform SEI. Notably, the WS_2_‐modified potassium metal battery achieves stable cycling at a high rate of 36 C for over 3000 cycles (Figure 21d).^[^ ^173^ ^]^ Furthermore, a novel high‐entropy SEI, rich in inorganic components, was constructed via in situ electrochemical conversion at the Sn_3_O_4_/Sn_2_S_3_ interface layer supported on the porous scaffold (Figure 21e). The in situ generated high‐entropy SEI layer exhibits low surface roughness, low surface potential, rapid potassium‐ion transport capability, and excellent mechanical properties. The K‐SnOS@NF||PTCDA full cell demonstrates a minimal capacity decay rate of merely 0.011% per cycle after 1650 cycles at a rate of 10 C (Figure 21f).^[^ ^56^ ^]^

### 4.3 Wide Temperature Range Battery

SEI design directly impacts battery performance under high and low temperatures.^[^ ^174^, ^175^, ^176^ ^]^ At low temperatures, electrolyte viscosity increases and ion migration slows. An optimal SEI design can reduce ion transport resistance at low temperatures, thereby enhancing low‐temperature performance. An SEI with specific composition and structure maintains good flexibility at low temperatures, resisting brittleness as temperature decreases to ensure unimpeded ion migration. In high‐temperature environments, the SEI requires excellent thermal stability to prevent decomposition and subsequent performance degradation caused by elevated temperatures. A well‐designed SEI can effectively isolate electrode materials from electrolytes, inhibiting excessive electrolyte decomposition and high‐temperature side reactions of electrode materials—thus extending battery lifespan under high‐temperature conditions.

At low temperatures, organic components with long‐chain structures in the SEI can maintain adequate flexibility, alleviating channel blockage caused by low‐temperature volume shrinkage to some extent. Additionally, inorganic components in the SEI enhance interfacial stability and facilitate ion transport. At high temperatures, the SEI prevents electrolyte decomposition and electrode‐electrolyte reactions primarily through its heat‐stable components (e.g., high‐temperature‐resistant inorganic compounds). These components form a stable protective layer, reducing heat‐induced chemical reactions within the battery.

**Figure 22:**
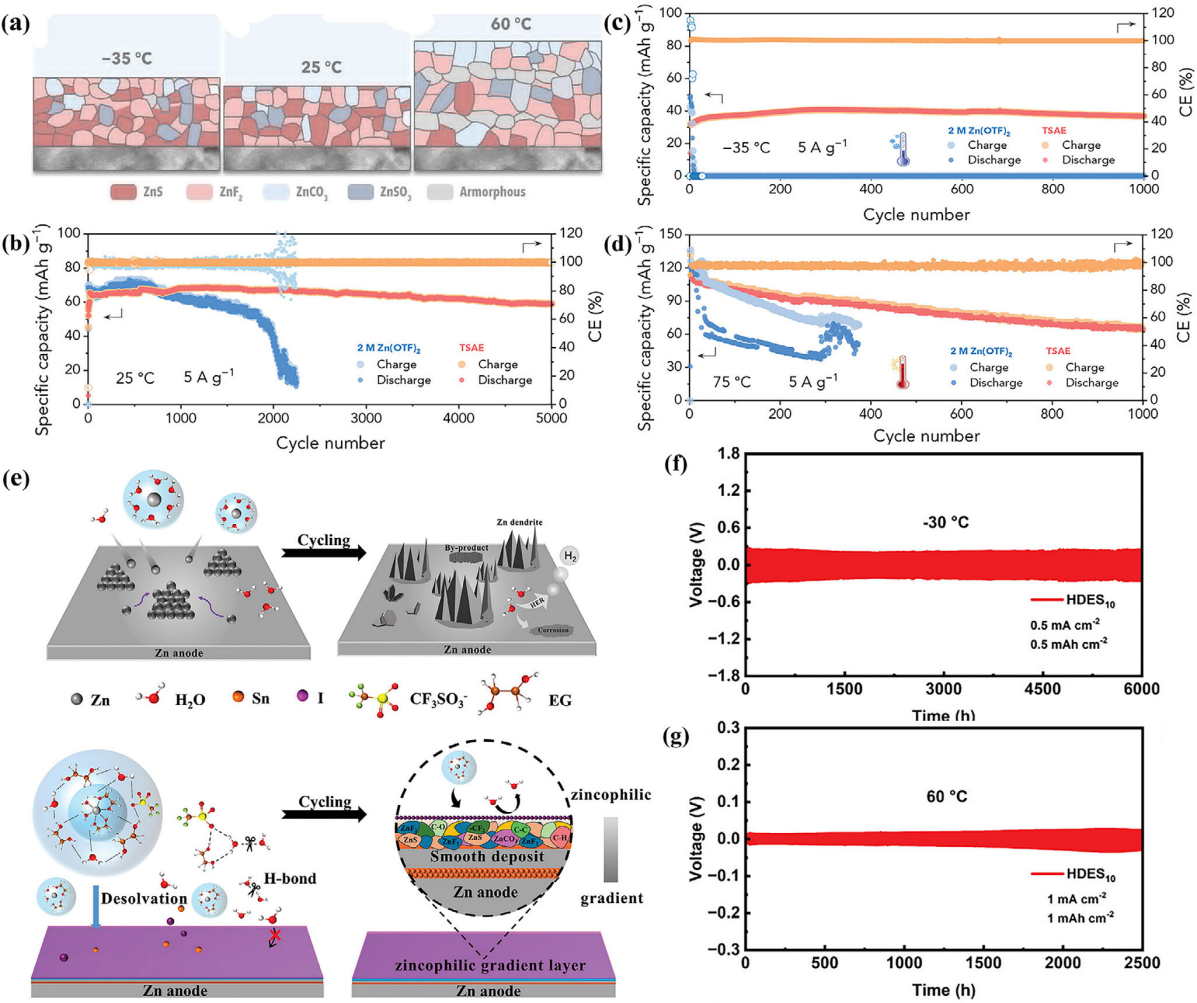
a) Schematics of the SEI configurations at different temperatures. b–d) Cycling stability of the full battery at different temperatures: 25, −35, and 75 °C.^[^ ^176^ ^]^ Copyright 2024, John Wiley and Sons. e) Schematic representation of the solvation structure of Zn^2+^ and the chemical behavior of the Zn anode interface in different electrolytes. f,g) Long cycle performance plots of Zn|Zn symmetric cells at −30 and 60 °C for HDES_10_.^[^ ^177^ ^]^ Copyright 2024, John Wiley and Sons.

Inspired by this design concept, a temperature‐adaptive electrolyte (TSAE) has recently been developed based on the principle of cosolvent and anion competitive coordination.^[^ ^176^ ^]^ This electrolyte exhibits dynamic solvation capability, generating an inorganic‐rich SEI at low temperatures and an SEI containing organic alkyl ethers and alkyl carbonates at high temperatures (**Figure** **22**a). When applied in PANI|Zn full batteries, TSAE demonstrates significant improvements, including excellent CE and ultra‐stable cycling performance over a wide temperature range (−35 to 75 °C) (Figure 22b–d). Additionally, Chen et al. proposed a novel hydrated deep eutectic solvent electrolyte synthesized from ethylene glycol and SnI_4_, which endows batteries with an excellent cycle life across the temperature range of ‐30 to 60 °C.^[^ ^177^ ^]^ Due to dissociation and reduction reactions between eutectic molecules and SnI_4_, a zincophilic gradient organic‐inorganic hybrid SEI forms on the Zn surface (Figure 22e), effectively inhibiting hydrogen evolution and regulating oriented Zn deposition. Zn|Zn symmetric batteries using this electrolyte exhibit remarkable cycling stability: over 7800 h at room temperature, over 6000 h at ‐30 °C, and over 2500 h at 60 °C (Figure 22f,g).^[^ ^177^ ^]^

**Figure 23:**
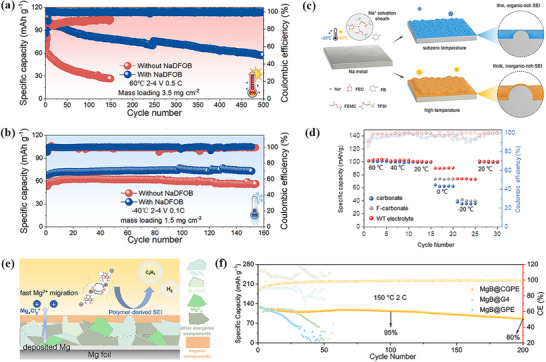
Long‐term cycling performance of Na||NFM batteries with BE and BE‐NaDFOB electrolytes at 60 °C a) and −40 °C b).^[^ ^127^ ^]^ Copyright 2025, Elsevier. c) Schematic illustration of Na growth behavior and temperature‐responsive SEI in the WT electrolyte at subzero and high temperature. d) Temperature‐dependent performance of Na||NVP cells ranging from −20 to 60 °C at 0.5 C.^[^ ^178^ ^]^ Copyright 2022, Elsevier. e) Schematic diagram of the polymer‐derived SEI. (f) Cycling stability of Mo_6_S_8_||MgB@CGPE||Mg battery at 150 °C, 2 C and the corresponding CE (1 C = 128.8 mAh g^−1^).^[^ ^179^ ^]^ Copyright 2025, John Wiley and Sons.

In sodium metal batteries, the introduction of a novel electrolyte additive—sodium difluoro‐oxalate borate—exerts a regulatory effect on the solvation structure of the electrolyte. This additive promotes the formation of uniform SEI films on both the cathode and anode surfaces, with its decomposition mechanism facilitating rapid desolvation of Na⁺ ions. Ultimately, these synergistic effects lead to the construction of a high‐ion‐conductivity SEI, which significantly enhances the overall stability of the battery. Notably, the additive demonstrates a profound impact on the performance of the NFM111||Na battery, enabling it to exhibit excellent rate capability, long‐term cycling stability, and favorable wide‐temperature‐range adaptability (**Figure** **23**a,b).^[^ ^127^ ^]^ Furthermore, the in situ construction of a novel fluorinated carbonate‐based electrolyte enables the formation of a temperature‐responsive solid electrolyte interphase (SEI) layer. (Figure 23c). The full cell assembled with this novel electrolyte enables stable cycling over a temperature range of −20–60 °C. (Figure 23d).^[^ ^178^ ^]^ In magnesium metal batteries, the incorporation of a multifunctional diamine additive enables in situ cross‐linking of the polymer electrolyte while facilitating the generation of abundant quaternary ammonium ions. Quaternary ammonium groups exhibit low reductive stability but exhibit high binding energy with the magnesium anode, thereby tending to adsorb onto the anode surface of magnesium metal batteries. This preferential adsorption promotes their selective decomposition, leading to the localized formation of a stable SEI (Figure 23e). At an elevated temperature of 150 °C, the assembled Mo_6_S_8_||MgB@CGPE||Mg full cell achieved stable cycling for 200 cycles, maintaining a capacity retention rate of 80% (Figure 23f).^[^ ^179^ ^]^

## 5 Conclusion and Prospect

### 5.1 Research Summary

This review systematically summarizes recent advances in electrolyte engineering and rational electrode‐electrolyte interfacial design for lithium, zinc, sodium, potassium, and magnesium metal batteries, with a particular focus on regulating the SEI via superwetting strategies. By analyzing the formation mechanisms, structural characteristics, and functional modulation of the SEI, as well as the roles of electrolyte additives and artificial SEI layers, this work clarifies the critical role of interfacial engineering in optimizing battery performance.

For lithium metal batteries, constructing a lithiophilic‐lithiophobic gradient SEI has emerged as a core strategy. Inorganic additives (e.g., SnF_2_, Cu(NO_3_)_2_) induce the formation of LiF/Li_3_N‐rich inorganic layers with high mechanical strength and rapid Li⁺ diffusion, while organic additives (e.g., EVS+FEC) and organic‐inorganic hybrid additives (e.g., AgTFSI) regulate SEI flexibility and ion transport. These designs effectively guide uniform Li deposition, suppress dendrite growth, and improve cycle stability and CE, as evidenced by enhanced performance in PEO‐based all‐solid‐state batteries and high‐voltage lithium metal batteries. In zinc metal batteries, constructing zincophilic‐zincophobic bilayer interfaces addresses challenges such as dendrite growth and side reactions. Eutectic electrolyte systems (e.g., ZnCl_2_‐SnCl_2_, Zn(ClO_4_)_2_‐EG‐InCl_3_) in situ form hybrid SEI layers: zincophilic components (e.g., Sn, In‐Mg alloys) reduce nucleation barriers, while zincophobic components (e.g., Zn_5_(OH)_8_Cl_2_·H_2_O, ZHS) block dendrite penetration. Additives like SN and AS further optimize SEI wettability and dynamic self‐repair, enabling ultra‐long cycles (over 3000 cycles) and wide‐temperature operation (−50 to 50 °C). For sodium metal batteries, sodiophilic‐sodiophobic SEI regulation relies on electrolyte additives (e.g., PFB, NaDFOB) and artificial layers (e.g., Ag‐modified layers). These strategies promote uniform Na deposition by reducing nucleation overpotential (via sodiophilic sites like Ag) and inhibit dendrites through NaF‐rich sodiophobic barriers, significantly extending cycle life (over 350 h for symmetric cells). In potassium metal batteries, bifunctional additives (e.g., ADN) and KF‐rich artificial SEI layers enhance stability. ADN preferentially decomposes to form potassiophilic C≡N groups, facilitating uniform K deposition and stabilizing cathodes, while KF‐rich SEI inhibits electrolyte decomposition—enabling stable cycles in full cells. For magnesium metal batteries, magnesiophilic‐magnesiophobic SEI is achieved via In/MgCl_2_ hybrid layers and boron‐based additives (e.g., TFEB). These designs reduce Mg nucleation barriers and form dense inorganic barriers (e.g., MgF_2_), supporting long cycles (over 1500 cycles) and high‐rate performance.

Furthermore, SEI design under extreme conditions—including high voltage, fast charging, and wide temperature ranges—is critical. High‐voltage batteries benefit from LiF‐rich SEIs to resist oxidation; fast‐charging systems require SEIs with low Li⁺ diffusion barriers (e.g., via the ISPN additive); and wide‐temperature batteries rely on temperature‐adaptive SEIs (e.g., enabled by the TSAE electrolyte) to maintain flexibility and stability across the range of ‐35 to 75 °C. Despite these advances, challenges persist: the action mechanisms of certain additives remain unclear, scaling up technologies is difficult, and SEI stability under complex extreme conditions is insufficient. These issues warrant further investigation.

### 5.2 Future Outlook

**Figure 24:**
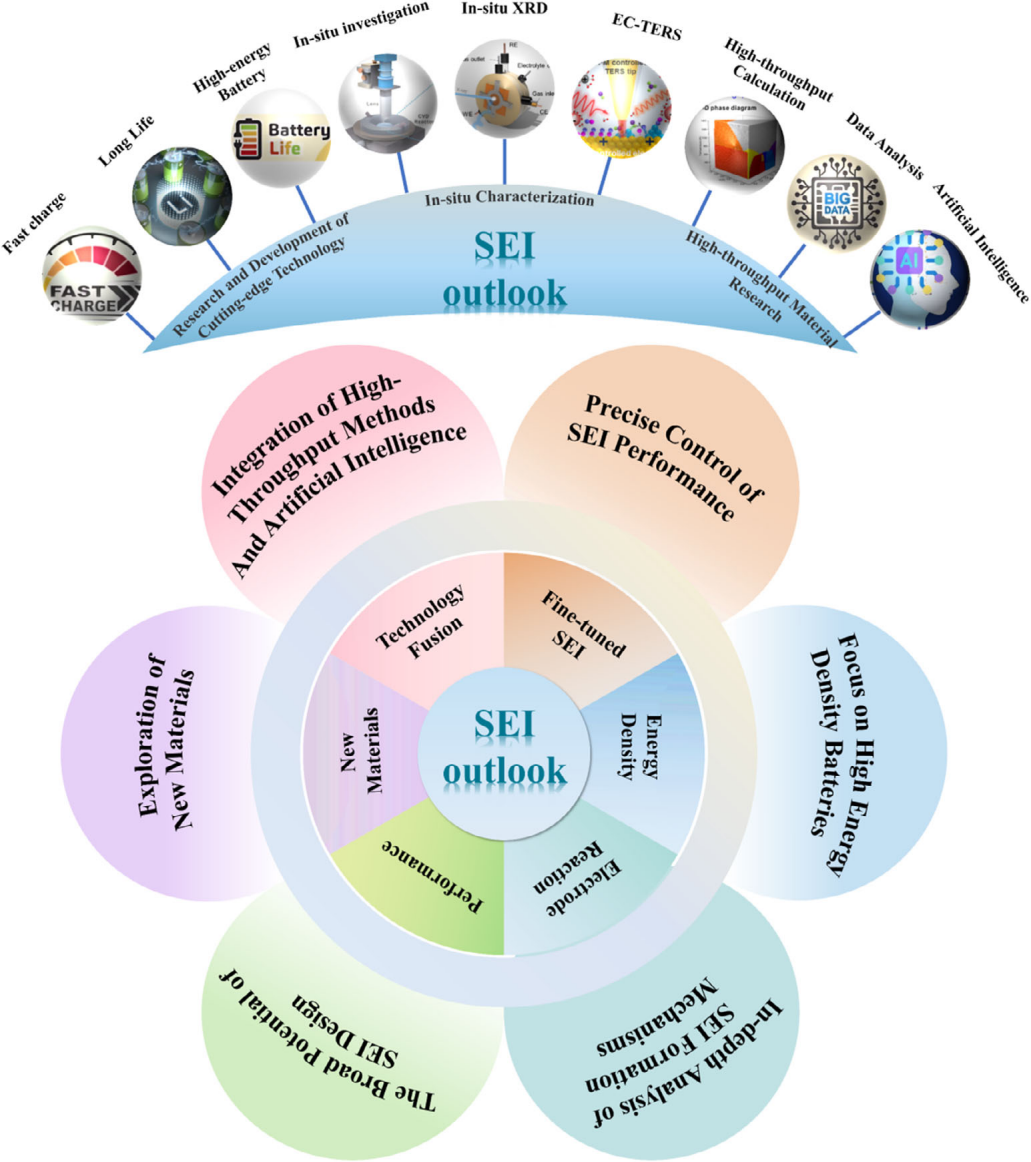
SEI Future vision.

Future research on SEI in batteries will advance in multiple directions (**Figure** **24**), each holding the potential to reshape the landscape of battery technology.

At the microscopic mechanism exploration level, achieving breakthroughs in key battery performances such as high energy density and long cycle life demands an in‐depth dissection of SEI formation mechanisms, chemical compositions, and structural evolution at the atomic and microscopic scales. This is especially crucial for metal anodes in emerging rechargeable battery, whose unique interfacial reaction kinetics and thermodynamics render SEI behavior more complex. Take lithium metal anodes as an example; the unstable interface caused by dendrite growth severely restricts battery safety and lifespan. Only by analyzing the dynamic changes of SEI during different charge‐discharge stages can key strategies for dendrite suppression be identified. This necessitates the utilization of advanced in situ characterization techniques. Dynamic electrochemical impedance spectroscopy (DEIS) can monitor the dynamic changes of SEI film resistance and ion diffusion impedance in real‐time, revealing the interfacial reaction kinetics; in situ transmission electron microscopy (TEM) provides a direct view of the nanoscale structural evolution of SEI, capturing its atomic‐level reconstruction details; and the electrochemical process visualization system based on cryoelectron tomography (cryo‐ET) to visualize the metal anode in 3D at nanometer resolution to allows researchers reveal atomic details in local areas. In the field of material innovation, beyond the material systems discussed in this perspective, emerging materials will inject new vitality into SEI engineering and interface design. The development of novel solvents has drawn significant attention. For instance, fluorinated solvents, with their high oxidation stability and low freezing point, can broaden the battery's operating temperature range while influencing the composition and structure of SEI. Multifunctional additives have become “magic molecules” for SEI regulation. They can not only act as film‐forming agents to construct a stable SEI on the electrode surface but also serve as redox mediators to enhance the battery's cycling stability. Additionally, research on new lithium salts and high‐concentration electrolyte systems is making continuous breakthroughs. The unique solvation structure in high‐concentration electrolytes can induce the formation of a LiF‐rich SEI film, significantly improving the stability of the electrode interface and ionic conductivity. In‐depth investigations into the interaction mechanisms between these materials and electrodes will provide new ideas for SEI regulation under extreme conditions (such as high and low temperatures, high‐rate charging and discharging), facilitating a qualitative leap in battery performance.

In terms of technology transfer, applying SEI research findings to next‐generation high‐energy‐density battery systems, including lithium metal batteries, zinc metal batteries, and all‐solid‐state batteries, is the key to solving interfacial stability challenges and promoting battery commercialization. In all‐solid‐state batteries, the compatibility between SEI and solid electrolytes directly affects the battery's interfacial impedance and cycling performance. By drawing on SEI regulation strategies from liquid batteries and combining them with the characteristics of solid electrolytes, developing suitable interface modification methods is expected to overcome the technical bottlenecks of all‐solid‐state batteries.

Regarding research method innovation, an interdisciplinary research paradigm integrating high‐throughput experiments, machine learning, and simulation calculations will become the mainstream. High‐throughput experimental techniques can rapidly synthesize and screen a large number of material combinations, generating vast amounts of experimental data. Machine learning algorithms can then mine hidden patterns from complex data, constructing SEI performance prediction models to achieve rapid optimization of materials and electrolyte formulations. First‐principles calculations and molecular dynamics simulations, on the other hand, can theoretically reveal the essence of SEI formation and evolution, guiding experimental research. The AI‐based inverse design method, through learning from extensive literature and experimental data, enables the intelligent design of battery interface materials and structures, significantly shortening the R&D cycle and opening up new dimensions for SEI research.

The research field of the SEI is vast. Its rich chemical compositions and diverse design space make it an attractive frontier in the energy storage domain. With the continuous integration of multiple disciplines and the innovation of research methods, SEI research is expected to yield more groundbreaking results, providing a powerful impetus for the development of new energy technologies.

## Conflict of Interest

The authors declare no conflict of interest.
